# A computationally designed ACE2 decoy has broad efficacy against SARS-CoV-2 omicron variants and related viruses in vitro and in vivo

**DOI:** 10.1038/s42003-023-04860-9

**Published:** 2023-05-12

**Authors:** Brandon Havranek, Graeme Walker Lindsey, Yusuke Higuchi, Yumi Itoh, Tatsuya Suzuki, Toru Okamoto, Atsushi Hoshino, Erik Procko, Shahidul M. Islam

**Affiliations:** 1 Department of Chemistry, University of Illinois at Chicago, Chicago, IL 60607 USA; 2 Sidney Kimmel Medical College at Thomas Jefferson University, Philadelphia, PA 19107 USA; 3 ComputePharma, LLC., Chicago, IL USA; 4 Department of Biochemistry, University of Illinois, Urbana, IL 61801 USA; 5 Department of Cardiovascular Medicine, Graduate School of Medical Science, Kyoto Prefectural University of Medicine, Kyoto, 602-8566 Japan; 6 Institute for Advanced Co-Creation Studies, Research Institute for Microbial Diseases, Osaka University, Osaka, Japan; 7 Cyrus Biotechnology, Inc., Seattle, WA USA; 8 Department of Chemistry, Delaware State University, Dover, DE 19901 USA

**Keywords:** Recombinant protein therapy, Protein design, Computational biophysics

## Abstract

SARS-CoV-2, especially B.1.1.529/omicron and its sublineages, continues to mutate to evade monoclonal antibodies and antibodies elicited by vaccination. Affinity-enhanced soluble ACE2 (sACE2) is an alternative strategy that works by binding the SARS-CoV-2 S protein, acting as a ‘decoy’ to block the interaction between the S and human ACE2. Using a computational design strategy, we designed an affinity-enhanced ACE2 decoy, **FLIF**, that exhibited tight binding to SARS-CoV-2 delta and omicron variants. Our computationally calculated absolute binding free energies (ABFE) between sACE2:SARS-CoV-2 S proteins and their variants showed excellent agreement to binding experiments. **FLIF** displayed robust therapeutic utility against a broad range of SARS-CoV-2 variants and sarbecoviruses, and neutralized omicron BA.5 in vitro and in vivo. Furthermore, we directly compared the in vivo therapeutic efficacy of wild-type ACE2 (non-affinity enhanced ACE2) against **FLIF**. A few wild-type sACE2 decoys have shown to be effective against early circulating variants such as Wuhan in vivo. Our data suggest that moving forward, affinity-enhanced ACE2 decoys like **FLIF** may be required to combat evolving SARS-CoV-2 variants. The approach described herein emphasizes how computational methods have become sufficiently accurate for the design of therapeutics against viral protein targets. Affinity-enhanced ACE2 decoys remain highly effective at neutralizing omicron subvariants.

## Abstract (web summary)

A computational design strategy is used to develop an affinity-enhanced ACE2 decoy, which is shown to be effective at neutralizing omicron subvariants in vitro and in vivo.

## Introduction

The severe acute respiratory syndrome coronavirus 2 (SARS-CoV-2), the causative agent of COVID-19 disease, has had profound implications on the global scale including over half a billion confirmed cases, 6 million deaths^1^ and long-term economic impact^2^. The scientific community was quick to rally together and a number of vaccines, monoclonal antibodies, and small molecules were approved or granted Emergency Use Authorization (EUA) by the U.S Food and Drug Administration and other agencies around the world for the prevention and treatment of COVID-19^3,4^. However, the SARS-CoV-2 RNA genome is rapidly evolving^5^ and a number of mutations in the spike (S) protein, the target of many vaccines and therapeutics, continue to appear leading to vaccine and monoclonal antibody resistance^6–8^. The B.1.617.2 (delta) variant first detected in India and the B.1.1.529 (omicron) variant first detected in Botswana were for many months variants of concern (VOC) in the United States^9^. The delta variant contains 12 mutations in its S protein, is roughly 60% more transmissible than the alpha variant^10^, and exhibits reduced neutralization by certain monoclonal antibodies and vaccines^11,12^. The omicron variant (B.1.1.529) and its sublineages are currently the dominant variants in the United States accounting for 100% of COVID-19 cases^9^. The omicron variant contains over 26 mutations in its S protein, with ~15 mutations located in the receptor-binding domain (RBD), a critical target for many therapeutic monoclonal antibodies. Omicron variants escape neutralization by monoclonal antibodies, including a cocktail of REGN10987 (imdevimab) and REGN10933 (casirivimab) and decreased neutralization by vaccinated and convalescent sera^7,13^. The omicron subvariant, BA.2, is more pathogenic and transmissible than the original BA.1 lineage, differs by 26 mutations from BA.1, and escaped 17 of 19 antibodies in preclinical and clinical developemet^14,15^. Even LY-CoV1404 (bebtelovimab), an EUA monoclonal antibody that maintained potency against earlier omicron variants, has reduced efficacy against newer omicron sublineages^15,16^. While the subvariant BA.2 and its lineages (i.e., BA.2.12.1) were, at one point, the large majority of SARS-CoV-2 infection worldwide, BA.5 then became the dominant strain in the United States and around the world and has now subsided to a ‘variant soup’^17^. Therefore, there is an urgent need to develop broad-spectrum pan-coronavirus therapeutics that can protect against both future SARS-CoV-2 variants and developing SARS-associated viruses that can cross over from animals to humans in the future^18^.

The S protein receptor-binding domain (RBD), located in the S1 subunit of the S protein, binds the human angiotensin-converting enzyme 2 (hACE2) leading to S1 shedding and proteolytic processing of S2 that is important for membrane fusion and release of viral RNA^19^. Various neutralizing therapeutics including protein minibinders^20,21^, peptides^22–24^, monoclonal antibodies^25^, and nanobodies^26^ have been developed to block the critical interaction between the RBD and hACE2. However, these therapeutics are often developed against the S protein of wild type or a specific variant of SARS-CoV-2, making them highly susceptible to mutational escape^27,28^. A strategy employed by our group^29,30^ and others^31–34^ includes using sACE2_2_ (soluble dimeric ACE2 that contains both the protease and dimerization domains) with enhanced S RBD affinity to outcompete native ACE2 expressed on host cells, acting as a ‘decoy’ to block the interaction between the RBD and hACE2. These sACE2 derivatives maintain close similarity to the native receptor making them extremely resistant to virus escape^32,35,36^. Any mutation in the RBD that limits binding to the sACE2 derivative will likely have reduced binding towards native ACE2 receptors potentially making the virus unfit to propagate. A previously engineered soluble ACE2_2_.v2.4-IgG1 decoy^31^ (with mutations T27Y, L79T, and N330Y) showed the ability to significantly mitigate lung injury and mortality in K18-hACE2 mice infected with SARS-CoV-2 WA-1/2020 and P.1 (Brazil) variants when administered intravenously^37^. Recently, ACE2_2_.v2.4-IgG1 was also shown to increase survival and mitigate lung injury in K18-hACE2 transgenic mice infected with the deadly P.1/gamma variant when administered via inhalation showcasing the possibilities of sACE2 to be administered via different mechanisms (i.e., intravenous and inhalation) for both prophylactic and therapeutic regimens^38^. Another engineered ACE2 decoy, 3N39v4, significantly reduced mortality and exhibited a therapeutic effect in hamster and hACE2 transgenic mice infected with omicron BA.1^36^. In addition, engineered soluble ACE2 decoys have shown the ability to neutralize previous coronaviruses (e.g., SARS-CoV-1), SARS-CoV-2 and its variants, and ‘pre-emergent’ sarbecoviruses (i.e., those that might cause human disease in the future) that use ACE2 as an entry receptor, showcasing soluble ACE2 decoys potential as a pan-coronavirus inhibitor^35,36,39^.

Using computational protein design, we previously engineered a four mutation sACE2 decoy (**FFWF)** with 1.8 nM affinity and ~10-fold tighter binding to the S protein compared to wild type sACE2^29^. Building upon our previous work, we used the Rosetta “Coupled Moves” flexible backbone design protocol^40^ to computationally design a 4 mutation sACE2 decoy **(FLIF)** with ~80-fold tighter binding and picomolar affinity for the delta variant compared to wild type sACE2. The computationally calculated binding free energies between sACE2:SARS-CoV-2 S proteins and its variants showed excellent agreement both qualitatively and quantitatively to flow cytometry, biolayer interferometry (BLI), and surface plasmon resonance (SPR) binding experiments. Our sACE2 decoy, **FLIF**, maintained tight binding to SARS-CoV-2 VOCs and showed the ability to potently neutralize SARS-CoV-2 delta, omicron (i.e., BA.1, BA.2, and BA.4/5), SARS-CoV-1, and other sarbecoviruses that have yet to emerge in a pseudotyped virus neutralization assay, pointing to the potential of the **FLIF** ACE2 receptor decoy as a pan-coronavirus therapeutic. Furthermore, **FLIF** neutralized authentic omicron BA.5 virus in vitro and showed a therapeutic benefit in Syrian hamsters infected with omicron BA.5. To the best of our knowledge, **FLIF** is one of the most potent computationally designed ACE2 decoys to date.

## Results

### Second-generation ACE2 decoy design

**Fig. 1:**
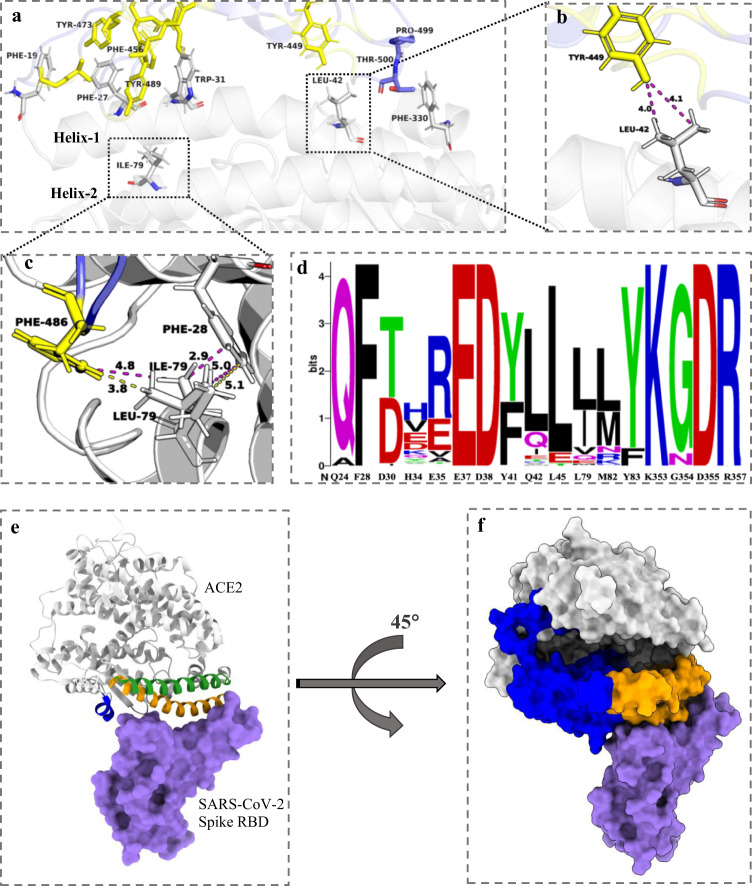
Computational design of second-generation ACE2 decoy (FLIF). **a** ACE2 residues F19, F27, W31, and F330 from the **FFWF** mutant and F27, L42, I79, F330 from the **FLIF** mutant are shown in gray. Hydrophobic RBD residues are shown in yellow with all other non-hydrophobic residues shown in purple. **b** Interface interaction distances between Tyr-449 (RBD) and Leu-42 (ACE2) in **FLIF** mutant represented in angstroms with magenta dashed lines. **c** Interface interaction comparison between Leu-79 in WT ACE2 and Ile-79 in **FLIF** ACE2 mutant. Distances for Leu-79 and Ile-79 to Phe-486 (RBD) and Phe-28 (ACE2) represented with yellow and magenta dashed lines, respectively. All distances are shown in angstrom. **d** Redesigned ACE2 residues shown as a sequence logo with wild type ACE2 amino acids shown on the x-axis. The height of the logo, in bits, indicates how many times an amino acid was preferred at that position from the top 10% of designs in 100 simulations using the Rosetta “Coupled Moves” flexible backbone design protocol. Sequence logo created using WebLogo from UC Berkeley^105^. **e** ACE2 (gray) and its binding motifs (helix 1: residues 19-53, orange; helix 2: residues 54-83, green; part of PD2 domain: residues 325-330, blue), targeted in the second-generation design of an ACE2 decoy, in complex with SARS-CoV-2 RBD (purple). **f** Protease domains (PD) of ACE2 PD1 (residues 19-102) and PD2 (residues 272-402) shown in orange and blue, respectively. The rest of ACE2 shown in gray and RBD shown in purple.

Using computational protein design with the Rosetta flex ddG protocol^41^, we previously engineered a four mutation sACE2 decoy, **FFWF**, containing mutations S19F, T27F, K31W, and N330F with 1.8 nM affinity to the S protein (Fig. 1a)^29^.

The T27F mutation is engaged in a hydrophobic region of the RBD and forms pi-stacking interactions with RBD residues Y473, F456, and Y489^29^. The N330F mutation improves packing against the aliphatic portion of RBD-Thr500^29^. Both the F27 and F330 mutations are in ACE2 protease domains (PD) PD1 (residues 19-102) and PD2 (residues 272-402), respectively (Fig. 1f). In a second-generation design, we were interested in increasing the coverage of mutations spatially across the ACE2 interface by including mutations in PD1, on both ACE2 helices (helix 1: residues 19-53 and helix 2: residues 54-83), and PD2 domains (Fig. 1e). We decided to include the T27F and N330F mutations in our second-generation ACE2 design, since F27 is on ACE2 PD1 (helix 1) with 3 highly favorable pi-stacking interactions, while F330 covers the PD2 domain and creates a favorable CH/π interaction with minimal entropic or steric penalty due to the restricted RBD-Pro499 side chain (Fig. 1a, e)^29,42^. The S19F mutation due to its position on the ACE2 N-terminal periphery (limited interactions with the RBD) and K31W due to tryptophan’s potential solubility issues were discarded in our second-generation design.

Using the Rosetta “Coupled Moves” flexible backbone design protocol^40^, we redesigned the local environment around the T27F and N330F mutations. ACE2 residues within 5 Å of heavy atoms on the RBD interface were allowed to be redesigned (except S19, K31, F27 and F330 in ACE2) to all amino acids besides cysteine, while RBD residues (plus S19, K31, F27 and F330 in ACE2) could change rotamer and/or backbone conformations (“repacking”) to accommodate the newly mutated side chains. The top 10% of designs based upon the summed cross-interface pairwise interactions energies from 100 Rosetta simulations were selected for further evaluation (Fig. 1d).

The wild type amino acid at ACE2 positions 24, 28, 37, 38, 45, 83, 353, 354, 355, and 357 were heavily preferred, while residues 34, 42, 79,and 82 had a plethora of design choices in excellent agreement with a recent deep mutational scanning (DMS) experiment (Fig. 1d)^31^. For example, residues Q24, F28, L45, Y83, K353, G354, D355, and R357 in ACE2 were all predicted to prefer the original wild type residue consistent with DMS, while mutations H34V, Q42L, L79I, and M82L in ACE2 were enriched in both the predictions by Rosetta and the DMS experiment. To create the second-generation ACE2 decoy, we chose mutation Q42L on ACE2 helix 1 due to its ability for leucine to form hydrophobic interactions with Y449-RBD and the mutation is spatially separated across the interface from mutations T27F and N330F (Fig. 1a, b). In addition, mutation L79I, in ACE2 helix 2, was chosen since it is an isomer of leucine whose sec-butyl side chain orientation can improve hydrophobic packing with F28 in ACE2 helix 1 to stabilize both ACE2 helix 1 and helix 2 and its binding with the RBD (Fig. 1a, c). Moreover, L79I directly engages F486-RBD with a hydrophobic interaction. The second-generation ACE2 decoy with 4 mutations (T27F, Q42L, L79I, and N330F) is referred to as **FLIF** throughout the manuscript.

### Computational and experimental verification of ACE2 decoy (FLIF) using free energy calculations and biolayer interferometry (BLI)

To verify whether **FLIF** had improved binding affinity over wild type ACE2 and our previously designed **FFWF** ACE2 decoy we used MD simulations to calculate the binding enthalpy using the molecular mechanics generalized Born surface area (MM/GBSA) method^43^. Briefly, four 100 ns MD replicates (i.e., using a different initial random velocity) were simulated for each wild type, **FFWF**, and **FLIF** ACE2 proteins with Wuhan RBD totaling 1.2 µs of simulation time.

**Fig. 2:**
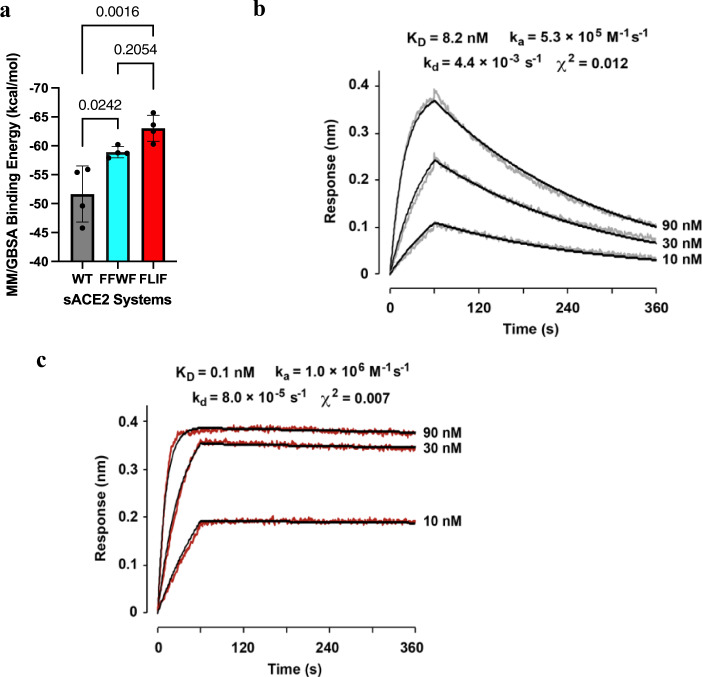
Computationally designed FLIF mutations increase affinity of sACE2-IgG1 for S of delta variant. **a** MM/GBSA computationally calculated binding free energies for wild type, **FFWF**, and **FLIF** sACE2 systems with Wuhan RBD. (Data are presented as the mean of MM/GBSA binding free energy values calculated from 4 independent MD simulations $\pm$ SD. *P* values were calculated by one-way ANOVA with a Tukey post hoc test. **b**, **c** BLI measurements of monovalent affinity between soluble delta RBD and immobilized sACE2-IgG1. Raw data for wild type ACE2 is gray (**b**) and for the **FLIF** mutant is red (**c**). Fitted curves are black. Concentrations of RBD are indicated at the right of sensorgrams. Association was from 0 to 60 s and dissociation was from 60 to 360 s.

The average binding enthalpy between wild type ACE2 and Wuhan RBD were −51.7 $\pm$ 4.2 kcal/mol, compared to −58.9 $\pm \,$0.83 kcal/mol and −63.0 $\pm$ 1.95 kcal/mol for **FFWF** and **FLIF**, respectively (Table 1 and Fig. 2a). Clearly, the MM/GBSA method overestimates the binding energy due to the disregard of entropy which is a major source of error and computational expense in MM/GBSA calculations^44^.

**Table 1:** FLIF is predicted to bind more strongly than FFWF to Wuhan RBD.

System	ΔG_MM/GBSA_ (kcal/mol)^a^	K_D_ (nM) from BLI Exp.^b^	ΔG_exp_ (kcal/mol)^c^	ΔG_CL-FEP_ (kcal/mol)^d^	Predicted K_D_ (nM)^e^
WT-ACE2/Wuhan RBD	−51.7 $\pm$ 4.2	16	−10.6	–	–
FFWF/Wuhan RBD	−58.9 $\pm \,$0.83	1.8	−11.9	–	–
FLIF/Wuhan RBD	−63.0 $\pm$ 1.95	–	–	–	–
FLIF/Wuhan RBD (CL-FEP)^f^	–	–	–	−12.4 $\pm$ 0.74^f^	0.77

Calculated binding free energies for wild type (Wuhan) SARS-CoV-2 S protein RBD with wild type ACE2 and ACE2 mutants (**FFWF** and **FLIF**).

^a^MM/GBSA calculated binding free energy calculated from the average of 4 independent 100 ns MD simulations $\pm$ SD.

^b^Experimentally determined K_D_ values from BLI (ref. ^29^).

^c^Experimental binding affinity converted using ΔG_exp_ = −RT ln(K_D_) with K_D_ values from (ref. ^29^).

^d^Computationally predicted absolute binding free energy calculated using CL-FEP.

^e^Predicted KD of the systems using the equation K_D_ = e^ΔGCL-FEP/RT^.

^f^FLIF-Wuhan RBD absolute binding affinity calculated using the CL-FEP approach^45^. The sampling was performed using 300 ns simulation time for each subsystem (complex, host, ligand, solvent) for 900 ns total. CL-FEP analysis was run 3 times using ρOSR = 3^45^. The reported values correspond to the mean and standard deviation among the 3 runs. The standard deviation (<1 kcal/mol) among the results from 3 independent runs of the CL-FEP analysis indicate the simulations are well converged.

We also set out to explore the absolute binding free energy (ABFE) between **FLIF**:Wuhan RBD using the CL-FEP approach^45^ since the MM/GBSA method is best utilized for calculating relative binding free energy (RBFE) values for comparison purposes^43,46^. CL-FEP combines both free energy perturbation (FEP) theory and central limit (CL) theory to reduce the large energetic noise of the potential energy distribution in MD simulations, allowing for the estimation of the ABFE change from explicit solvent simulations. CL-FEP has been applied recently on protein-protein^45,47^ and protein-ligand systems^45^, with results exceptionally similar to experimental binding affinities and those calculated using more demanding free energy approaches such as geometrical^48,49^ or alchemical pathways^50^. To calculate the ABFE using CL-FEP theory, we used 300 ns of MD simulations (i.e., 3 replica simulations of 100 ns each) for each of the complex (sACE2:RBD), receptor (sACE2), ligand (RBD), and solvent totaling 900 ns of simulation time for each system. The calculated ABFE for **FLIF**:Wuhan RBD was −12.4 kcal/mol (~K_D_ 0.77 nM) (Table 1). The predicted K_D_ calculated from the CL-FEP method shows increased affinity for **FLIF** compared to **FFWF** (Table 1) and suggests **FLIF** is worth examining experimentally.

There have been conflicting reports on whether the delta variant binds more strongly to ACE2 than wild type S. Some groups report very similar and even decreased binding affinity of the delta variant to ACE2^51,52^. Using biolayer interferometry (BLI), we measured the monovalent affinity for soluble RBD from delta to wild type ACE2 and measured a K_D_ of 8.2 nM (Fig. 2b) which is about 2-fold tighter than the measured K_D_ of 16 nM for wild type RBD from our recent report^29^. The ~2-fold greater affinity of delta RBD for wild type ACE2 align with the results from Mannar et al.^53^, Vogt et al.^54^, and Wang et al.^55^ who found 1.5, 2.15, and 2.12-fold increases, respectively. In addition, we used BLI to measure the monovalent affinity between our **FLIF** decoy and delta RBD (Fig. 2c). The measured K_D_ was 0.1 nM which constitutes an ~80-fold increase in binding compared with delta RBD:WT ACE2. These results showcase the predictive power of our computational approach and further highlight the ability for **FLIF** to broadly bind SARS-CoV-2 S variants with possibly even greater affinity than the ancestral variant, which is an intrinsic benefit of affinity enhanced ACE2 decoys^35^. Impressively, our computationally designed **FLIF** decoy bound delta RBD with similar affinity (K_D_ ~ 0.1 nM) to ACE2.v2.4 (K_D_ ~ 0.3 nM) which is one of the most efficacious ACE2 mutants reported to date^31,37^. It is worth noting that ACE2.v2.4 was designed after multiple rounds of experimental mutagenesis, while **FLIF** was constructed solely using a computational approach.

### Computationally designed ACE2 decoy (FLIF) broadly binds SARS-CoV-2 S proteins of VOCs

Furthermore, it has been shown that the Omicron BA.1 variant escapes antibodies due to the 15 mutations in its RBD, with 10 of the mutations in the receptor-binding motif (RBM) that interacts directly with ACE2^14,15,56^. However, it is less clear how these mutations impact the ability for BA.1 RBD to bind with ACE2. Some groups have reported that BA.1 binds ACE2 with lower affinity than WT (Wuhan) S^51,57,58^, while others report increased binding affinity to ACE2^52–54,59–61^. Therefore, we calculated the ABFE between WT ACE2:BA.1 RBD using the CL-FEP approach^45^.

**Fig. 3:**
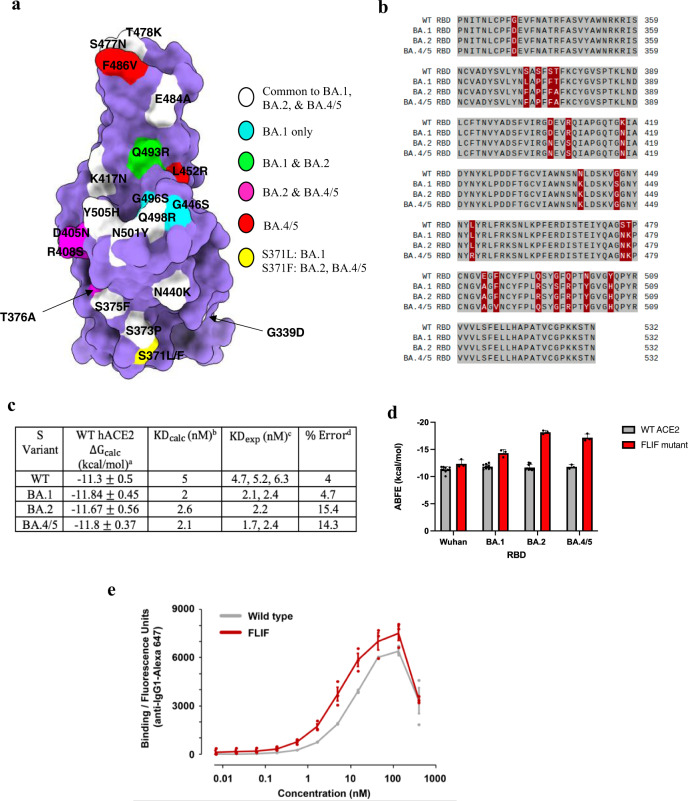
Computationally designed ACE2 decoy (FLIF) broadly binds SARS-CoV-2 S protein of VOCs. **a** Position of RBD mutations mapped on the surface. The RBD is represented as surface in purple with mutations common to BA.1, BA.2, and BA.4/5 in white, mutations unique to BA.1 in cyan, those unique to BA.1 and BA.2 in green, unique to BA.2 and BA.4/5 in pink, and mutations only in BA.4/5 in red. Residue 371 in yellow has a different mutation in both BA.1 and BA.2/BA.4/5. The RBD interface that binds with ACE2 (i.e., binding interface) is pointed out of the page towards the reader. **b** Sequence alignment between RBD of WT (PDB: 6M0J), BA.1 (7WBP), BA.2 (7ZF7), and BA.4/5 (7ZXU) SARS CoV-2 variants. Residues that are identical between all four RBDs are shown in gray. Non-conserved residues are shown in red. **c** Calculated CL-FEP absolute binding free energy values for RBD of WT, BA.1, BA.2, and BA.4/5 variants with wild type ACE2. ^a^The sampling was performed using 300 ns simulation time for each subsystem (complex, host, ligand, solvent) for 900 ns total each system. CL-FEP analysis was run 10 times using ρOSR = 3^45^ for each system. The reported values correspond to the mean and standard deviation (SD) among the 10 runs. The standard deviation (<1 kcal/mol) among the results from 10 independent runs of the CL-FEP analysis indicate the simulations are well converged. ^b^Predicted K_D_ of the systems using the equation K_D_ = e^ΔGcalc/RT^. ^c^Experimentally determined K_D_ values from SPR (ref. ^56^, ref. ^65^, ref. ^67^, ref. ^62^, and ref. ^59^). ^d^Percent error calculated between KD_calc_ and KD_exp_ using the closest value of KD_exp_ to our results. **d** Calculated CL-FEP absolute binding affinities for WT and **FLIF** ACE2 with WT, BA.1, BA.2, and BA.4/5 SARS-CoV-2 S RBDs. Individual data points shown plus the SD among 9-10 runs (for WT ACE2, besides WT-BA.4/5 (3 runs)) and 3 runs (for **FLIF**) of the CL-FEP analysis. All SD < 1 kcal/mol indicating the simulations are well converged. **e** Avid binding measured by flow cytometry of wild type (gray) and **FLIF** mutant (red) sACE2-IgG1 to cells expressing full length S of BA.2 omicron SARS-CoV-2. Data are mean ± SEM, *N* = 3 biological replicates.

The calculated absolute binding affinities were −11.3 kcal/mol (~K_D_ 5 nM) and −11.8 kcal/mol (~K_D_ 2 nM) for WT ACE2:WT RBD and WT ACE2:BA.1 RBD, respectively (Fig. 3c). We used PDB 6M0J which contains Wuhan (WT) RBD in complex with ACE2 to calculate the ABFE for the WT ACE2:WT RBD system^62^. In the same work in which the crystal structure of 6M0J was solved, the authors reported a binding affinity of −11.4 kcal/mol using SPR experiments within 0.1 kcal/mol of our computational prediction (Fig. 3c)^62^. Furthermore, Blazhynska and coworkers recently applied the rigorous geometrical transformations and potential of mean force (PMF) calculations on the same system used in our calculation (i.e., PDB: 6M0J) and calculated an ABFE of −11.5 kcal/mol^63^. Both the geometrical^63,64^ and the CL-FEP approach^45^ required a similar simulation time of 1.07 μs and 0.9 μs, respectively, and came to very similar and accurate results. Therefore, due to the accuracy of the CL-FEP approach to recapitulate the ABFE of both the rigorous geometric route and experiments, we consider it suitable to study the ABFE of SARS-CoV-2 spike RBD:ACE2 complexes.

Our calculated values suggest around a 2.5-fold increase in binding affinity for the BA.1 variant with wild type ACE2 compared to the ancestral Wuhan variant. These values are also in excellent quantitative agreement with the SPR experiments conducted by Wang et al.^56^ and Lan et al.^59^. For example, Wang et al.^56^ utilized the spike trimer with dimeric ACE2 in SPR experiments and calculated a K_D_ of 5.2 nM and 2.10 nM for WT S:ACE2 and BA.1S:ACE2, respectively. In addition, Lan et al.^59^ utilized SPR experiments using ACE2 and RBDs of both WT and BA.1, similar to the systems in our calculation, and calculated a K_D_ of 6.3 nM and 2.4 nM for WT RBD:ACE2 and BA.1 RBD:ACE2, respectively (Fig. 3c). These studies show the precision of the CL-FEP method to reproduce the absolute binding affinities of SARS CoV-2 S variants with ACE2 from experiments. On a qualitative scale, our estimation of a 2.5-fold increase of binding for BA.1 RBD:ACE2 compared to WT RBD:ACE2 is also in excellent agreement with Vogt et al.^54^, Yin et al.^61^, Mannar et al.^53^, Cameroni et al.^52^, and Meng et al.^60^ who found 2, 2, 1.4, 2.4, and 2.8-fold increases, respectively. The computationally predicted 2.5-fold increase for omicron BA.1 to WT ACE2 is very similar to the 2-fold increase in binding for the delta variant that we determined using BLI (Fig. 2b). Our computationally and experimentally calculated results agree exceptionally well to other BLI and SPR binding experiments with multiple groups reporting a similar ~2-fold increase in binding for both delta and omicron BA.1 variants to WT ACE2^53,54,65^, which suggests that delta and BA.1 variants have similar affinities for ACE2. Moreover, these results suggest that the 15 mutations in the BA.1 RBD have evolved to evade neutralizing antibodies without compromising spike affinity for host receptor ACE2.

We also calculated the ABFE for omicron subvariant BA.2. In the RBD, BA.1 contains unique mutations S371L, G446S, and G496S, while BA.2 carries S371F, T376A, D405N, and R408S. Mutations D405N and R408S close to the interface of ACE2 could potentially modulate BA.2 affinity for ACE2 (Fig. 3a, b). The calculated binding affinity of −11.6 kcal/mol (~K_D_ 2.6 nM) was only a slight decrease compared to that of BA.1 and indicate subvariants of omicron have not lost receptor affinity which suggest the omicron subvariants will still be highly susceptible to neutralization by the ACE2 decoys (Fig. 3c). The calculated value is also in agreement with the estimated K_D_ from Wang et al.^56^ of 2.2 nM. Furthermore, the ABFE calculated via the geometric approach by Chipot and colleagues also agree with our calculations within 0.2 kcal/mol^66^.

In addition to BA.2, omicron variants BA.4 and BA.5 (referred to as BA.4/5 due to their identical S sequences) have fueled a new wave of infections in the United States with international spread. Neutralization of BA.4/5 by triple dosed vaccine serum is reduced compared to BA.1 and BA.2^67^. Even more troubling, there are significant reductions in titers against BA.4/5 compared to BA.1 and BA.2 from sera in individuals who suffered vaccine breakthrough BA.1 infections^67^. This suggests the risk of reinfection in individuals already infected with early omicron subvariants has increased and that the omicron variant has continued to evolve with increasing neutralization escape^68^. In fact, 10/28 potent omicron specific monoclonal antibodies derived following vaccine breakthrough BA.1 infection are completely attenuated against BA.4/5, while others have large reductions in activity including commercial antibodies developed for clinical use^67^. BA.4/5 contains an additional 2 mutations from BA.2 including L452R, previously seen in the delta variant, and F486V, which are both close to the ACE2 interface (Fig. 3a). Furthermore, BA.4/5 lacks the Q493R mutation which is reverted to Q493 found in the original Wuhan variant (Fig. 3a, b). Therefore, it is imperative that ACE2 decoys maintain tight binding to BA.4/5. We calculated the ABFE for WT ACE2:BA.4/5 RBD which was −11.8 kcal/mol (~K_D_ 2.1 nM) in close agreement with the K_D_ of 1.7 nM calculated using SPR with BA.4/5 spike trimer and 2.4 nM using BA.4/5 RBD (Fig. 3c)^56,67^. On a qualitative scale, Cao et al.^65^ used SPR experiments with the RBDs of omicron variants and found the binding affinity for BA.4/5 to be very similar to BA.1 (i.e., within 0.1 nM) in excellent agreement with our computationally calculated values (Fig. 3c). Again, while omicron subvariants such as BA.4/5 continue to evolve to evade neutralization, their binding to host receptor ACE2 is not compromised. The epistatic effect of the Q498R and N501Y mutations in the omicron subvariants provides an affinity buffer which allows omicron S to tolerate mutations that individually decrease ACE2 binding but contribute to antibody escape^69^. Moreover, our results further show that the CL-FEP approach^45^ can capture the ABFE values for S protein variants with ACE2 in very close agreement with experimental binding assays (Fig. 3c).

Using BLI, we showed that our **FLIF** mutant bound the delta variant RBD with picomolar affinity (i.e., K_D_ 0.1 nM) and ~80-fold improvement over wild type ACE2 (Fig. 2c). We were also interested to see whether **FLIF** maintained tight binding to omicron and its subvariants (i.e., BA.1, BA.2, and BA.4/5) since they contain mutations in their RBD that differ substantially from delta and have evolved to evade the majority of monoclonal antibodies that are either FDA approved or in pre-clinical/clinical development^15^. We calculated the ABFE for **FLIF** to omicron BA.1, BA.2, and BA.4/5 RBDs and found that our decoy maintained tight binding (i.e., predicted K_D_ < 0.1 nM) with lower K_D_ values than those calculated with Wuhan RBD or with the WT ACE2 decoy binding to omicron subvariants (Fig. 3c, d). We note that the ABFE for **FLIF** with omicron subvariants are likely overestimated because the mutations in **FLIF** were modeled due to the lack of a crystal structure. Nevertheless, **FLIF** is still predicted to maintain extremely high affinity for spike RBDs of omicron BA.1, BA.2, and BA.4/5, consistent with other affinity enhanced ACE2 mutants that bind SARS-CoV-2 variants with greater affinity than wild type^37,39^.

To support our computational calculations, we used flow cytometry to measure avid binding of wild type and **FLIF** mutant sACE2-IgG1 to cells expressing full length S of BA.2 omicron SARS-CoV-2 (Fig. 3e). Although avid binding can mask differences in monovalent affinity^31,37,38^, the **FLIF** decoy clearly bound substantially more tightly to full length S of BA.2 omicron in comparison to wild type ACE2 (Fig. 3e). At higher concentrations (i.e., >100 nM) binding of sACE2 to BA.2S expressing cells decreased which suggests shedding of ACE2-bound S1 consistent with Zhang et al.^37^. The flow cytometry experiments further support our computational predications that **FLIF** is able to maintain tight binding to omicron variants.

### Computationally designed FLIF decoy broadly neutralizes SARS-CoV-2 variants including omicron BA.1, BA.2, and BA.4/5

**Fig. 4:**
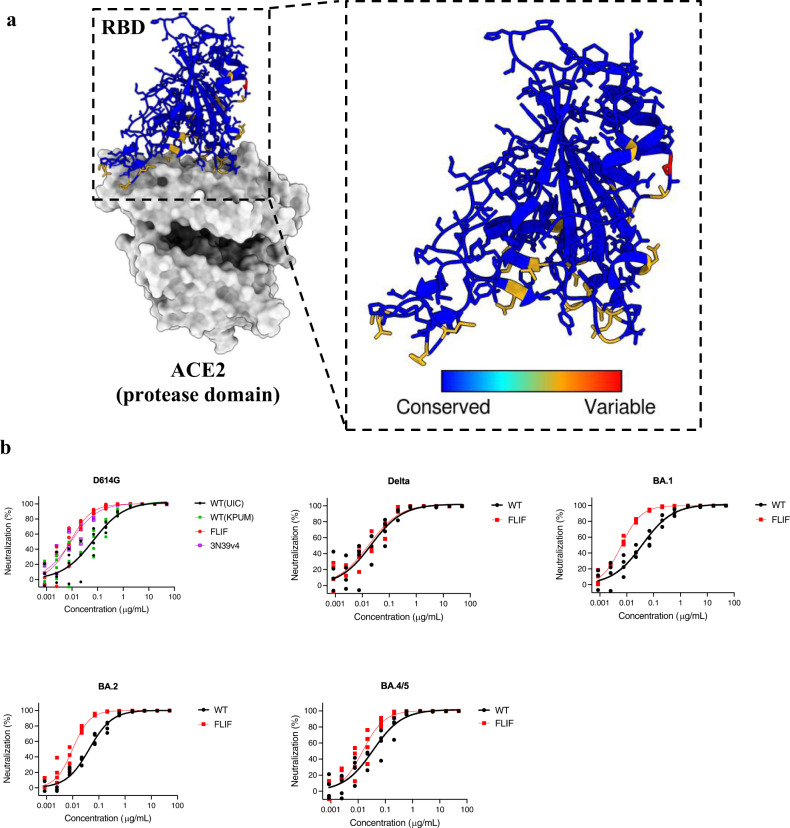
Computationally designed ACE2 decoy (FLIF) broadly neutralizes SARS-CoV-2 variants in a pseudovirus assay. **a** RBD of SARS-CoV-2 (PDB: 6M0J) colored by diversity between SARS-CoV-2 variants (D614G, delta, omicron BA.1, BA.2, and BA.4/5). Blue color indicates conserved regions while red indicates variable regions. **b** Neutralization efficacy in 293T/ACE2 cells is shown for **FLIF** and wild type ACE2 decoys against five SARS-CoV-2 variants. **FLIF** and WT(UIC) ACE2 decoys are fusions of ACE2 amino acids 18-732, containing the protease and collectrin-like dimerization domains, to human IgG1 Fc. Two independently cloned and purified WT ACE2 decoys were tested: UIC and KPUM. (*n* = 4 technical replicates, individual data points shown. Some data points overlap due to similar values on technical replicates).

Using CL-FEP, BLI, and flow cytometry we showed that the **FLIF** decoy can maintain extremely high affinity for SARS-CoV-2 VOCs including the omicron subvariants (Fig. 3d, e). Therefore, we evaluated the efficacy of our **FLIF** decoy to neutralize SARS-CoV-2 S variants D614G, delta, omicron BA.1, BA.2, and BA.4/5 in a pseudovirus assay (Fig. 4a, b).

Our **FLIF** decoy was first compared to ACE2 mutant 3N39v4^13,32^, a potent decoy designed using multiple rounds of experimental directed evolution (Fig. 4b). Impressively, the computationally designed **FLIF** mutant neutralized the D614G variant with similar efficacy to 3N39v4 (Table 2). Both **FLIF** and 3N39v4 showed greater neutralization in comparison to wild type ACE2 from two independent laboratories. Among the therapeutic antibodies authorized for clinical use, only bebtelovimab retains full potency for both BA.2 and BA.4/5^70^, although bebtelovimab’s potency is severely diminished against newer subvariants that are increasing in frequency^16^. While omicron continues to evolve to be more evasive to antibodies and vaccination, the **FLIF** decoy maintained potent neutralization efficacy against the three subvariants of omicron tested at concentrations more efficacious than wild type ACE2 (Fig. 4b and Table 2). The neutralization data supports our computational and experimental binding assays and reiterates that the **FLIF** decoy will likely continue to be efficacious against developing variants of SARS-CoV-2. For quantitative comparison, Table 2 contains IC_50_ values for all pseudovirus data.

**Table 2:** IC_50_ values for Wildtype and FLIF ACE2 decoys against SARS-CoV-2 (and its variants), SARS-CoV, and sarbecovirus pseudoviruses^a^.

	WT	FLIF	WT (KPUM)	3N39v4^b^
D614G	0.06769	0.007994	0.07431	0.00778
Delta	0.0241	0.01993	–	–
BA.1	0.03726	0.006439	–	–
BA.2	0.03988	0.01079	–	–
BA.4/5	0.03439	0.01415	–	–
SARS1	0.07545	0.01009	–	–
PG-GD-1	0.01649	0.03831	–	–
PG-GX-P5L	Unstable	0.1635	–	–
WIV1	0.02232	0.008895	–	–
RsSHC014	0.01209	0.01461	–	–

^a^All IC_50_ values are in μg/mL.

^b^ACE2 decoy 3N39v4 for comparison from refs. ^13,32^.

### Computationally designed FLIF decoy shows breadth of cross-neutralization against SARS-CoV and sarbecoviruses

**Fig. 5:**
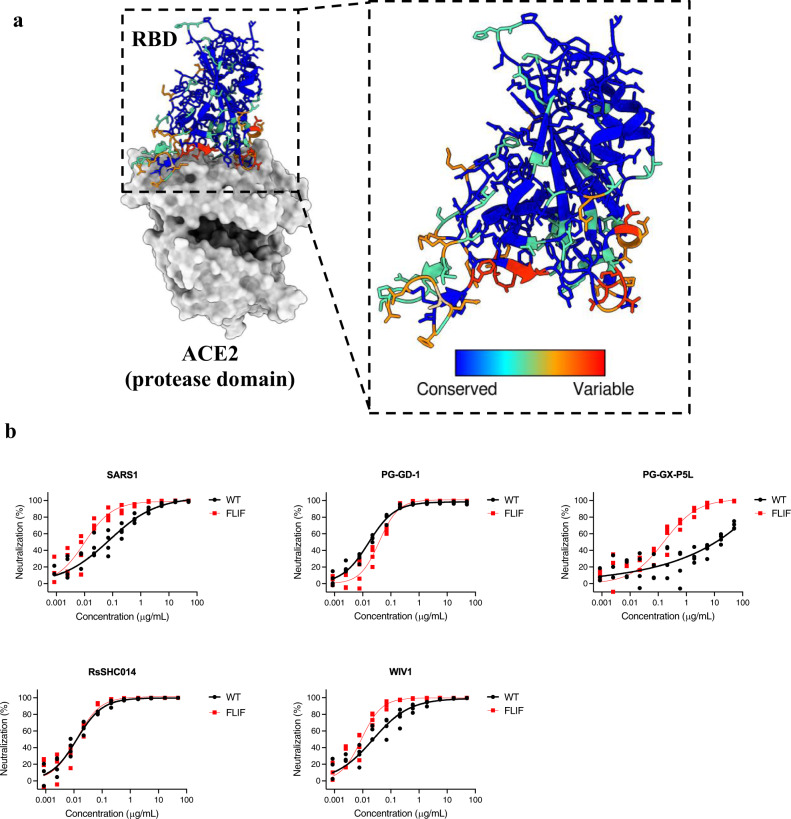
Computationally designed ACE2 decoy (FLIF) broadly neutralizes SARS-CoV and sarbecoviruses in a pseudovirus assay. **a** RBD of SARS-CoV-2 (PDB: 6M0J) colored by diversity between SARS-CoV and sarbecoviruses (WIV1, RsSHC014, GD-1, and GX-P5L). Blue color indicates conserved regions while red indicates variable regions. **b** Neutralization efficacy in 293 T/ACE2 cells is shown for **FLIF** and wild type ACE2 decoys against pseudoviruses expressing S of SARS-CoV and four sarbecoviruses. (*n* = 4 technical replicates, individual data points shown. Some data points overlap due to similar values on technical replicates).

When engineering ACE2 decoys for tight binding, there is a balance that needs to be maintained between tight affinity and breadth. To test the breadth of cross-neutralization we performed neutralization assays with **FLIF** and wild type ACE2 decoys against SARS-CoV and other sarbecoviruses that use ACE2 as their receptor (Fig. 5a, b), including three viruses from the SARS-CoV clade (SARS-CoV, WIV1, and RsSHC014) and two viruses from the SARS-CoV-2 clade (GD-1 and GX-P5L) (Table 2).

**FLIF** neutralized SARS-CoV, GX-P5L (pangolin), and WIV1 (bat) with greater efficacy than wild type ACE2 and GD-1 (pangolin) and RsSHC014 (bat) with similar efficacy to wild type ACE2 (Fig. 5b and Table 2). Notably, **FLIF** potently neutralized GX-P5L (pangolin) while wild type ACE2 showed much lower neutralization. Importantly, **FLIF** could potently neutralize pangolin (i.e., GD-1 and GX-P5L) and bat (i.e., WIV1 and RsSHC014) sarbecoviruses. These variants are seen as a risk for future zoonotic transmission^71,72^; thus, these results indicate that affinity enhanced ACE2 decoys have neutralization potency against a broad range of sarbecoviruses that could potentially cross over to humans in the future and potentially be used as a pan-coronavirus therapeutic. As seen in Fig. 5a, the sequence diversity between various sarbecoviruses differ substantially. However, these results indicate that **FLIF** is not over-engineered at the expense of breadth as **FLIF** is able to neutralize all sarbecoviruses tested.

### Computationally designed ACE2 decoy, FLIF, neutralizes authentic omicron BA.5 in vitro and confers protection against authentic BA.5 in vivo

**Fig. 6:**
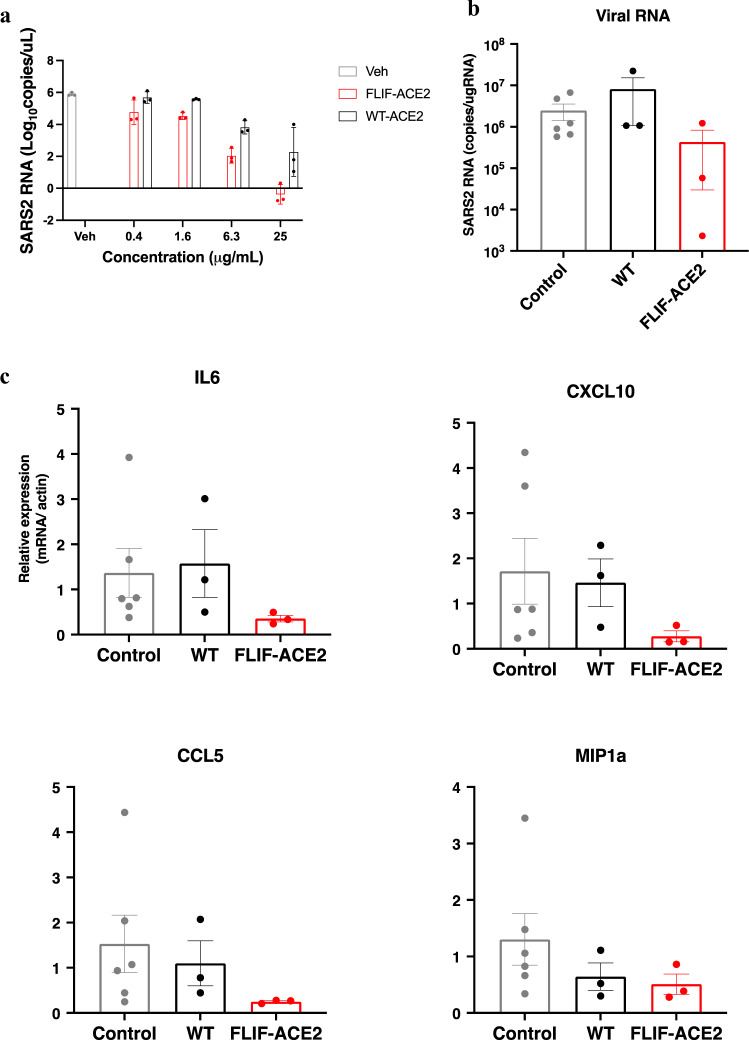
FLIF mutant potently neutralizes authentic omicron BA.5 in vitro and confers protection in hamsters. **a** Neutralization efficacy of **FLIF** and wild type ACE2 decoys was compared by infecting Vero E6/TMPRSS2 cells with authentic omicron BA.5 virus. RNA copy number was analyzed by qRT-PCR against nucleocapsid. *n* = 3 technical replicates. The decoys contain the extracellular protease and dimerization domains of ACE2 (residues 18-732) fused at the C-terminus to human IgG1 Fc. **b** Syrian hamsters were challenged with 10^4^ PFU omicron BA.5 via intranasal route and ACE2 decoys were administered 2 h later by intraperitoneal injection at 20 mg/kg. Quantification of viral RNA in the lungs of treated (with **FLIF** mutant or wild type ACE2 decoy) and untreated (control) Syrian hamsters at day 5 was performed by qRT-PCR against nucleocapsid (*n* = 6 in control and *n* = 3 in treatment groups). **c** Gene expression of inflammatory cytokines and chemokines at day 5 was quantified by qRT-PCR of lung tissue. The expression of β-actin was used for normalization (*n* = 6 in control and *n* = 3 in treatment group).

Next, we directly compared the effects of our **FLIF** mutant against the wild type ACE2 decoy on propagation of authentic omicron BA.5 in vitro and in vivo. Vero E6 cells expressing transmembrane protease serine 2 (TMPRSS2) were infected with omicron BA.5 in the presence of **FLIF** or wild type ACE2 (Fig. 6a).

At all concentrations tested (i.e., 0.4, 1.6, 6.3, and 25 μg/mL) **FLIF** was more efficacious than wild type ACE2 at neutralizing BA.5 (Fig. 6a) consistent with our pseudovirus data. Impressively, the **FLIF** mutant potently neutralized omicron BA.5 at concentrations of 6.3 and 25 μg/mL. Indeed, viral RNA was almost undetectable in cells treated with 25 μg/mL **FLIF**. Furthermore, we directly compared the therapeutic benefit of **FLIF** and wild type ACE2 decoys in Syrian hamsters. The hamsters were infected by the intranasal route with 1 × 10^4^ plaque-forming units (PFU) of omicron BA.5 and then treated with either **FLIF** or wild type ACE2 (20 mg/kg) by intraperitoneal route 2 h after inoculation. After 5 days, viral RNA in the lungs of the hamsters was suppressed by treatment with **FLIF** but not by wild type ACE2 (Fig. 6b). Moreover, the gene expression of inflammatory cytokines and chemokines IL6, CXCL10, and CCL5 showed a marked reduction in transcription by treatment with the **FLIF** mutant but not wild type ACE2 (Fig. 6c). These data indicate that **FLIF** is efficacious at neutralizing omicron BA.5 in vitro and in vivo and affinity enhanced ACE2 mutants such as **FLIF** hold a major advantage at neutralizing omicron subvariants over the non-affinity enhanced wild type ACE2.

### Atomistic rationale of affinity enhancement by FLIF decoy to BA.4/5 RBD

To provide an atomistic rationale of affinity enhancement for omicron BA.4/5S RBD to wild type and **FLIF** ACE2, we performed molecular dynamics (MD) simulations. By itself, the N501Y mutation present in the omicron subvariants improves binding to ACE2, while the Q498R mutation alone reduces affinity to ACE2^69,73^. However, the epistatic effect of both N501Y/Q498R combined in omicron and its subvariants enables omicron RBD to bind ACE2 around ~2-fold greater than Wuhan RBD despite having a large number of mutations that contribute to antibody escape but are deleterious for ACE2 binding (Fig. 3c)^69^.

**Fig. 7:**
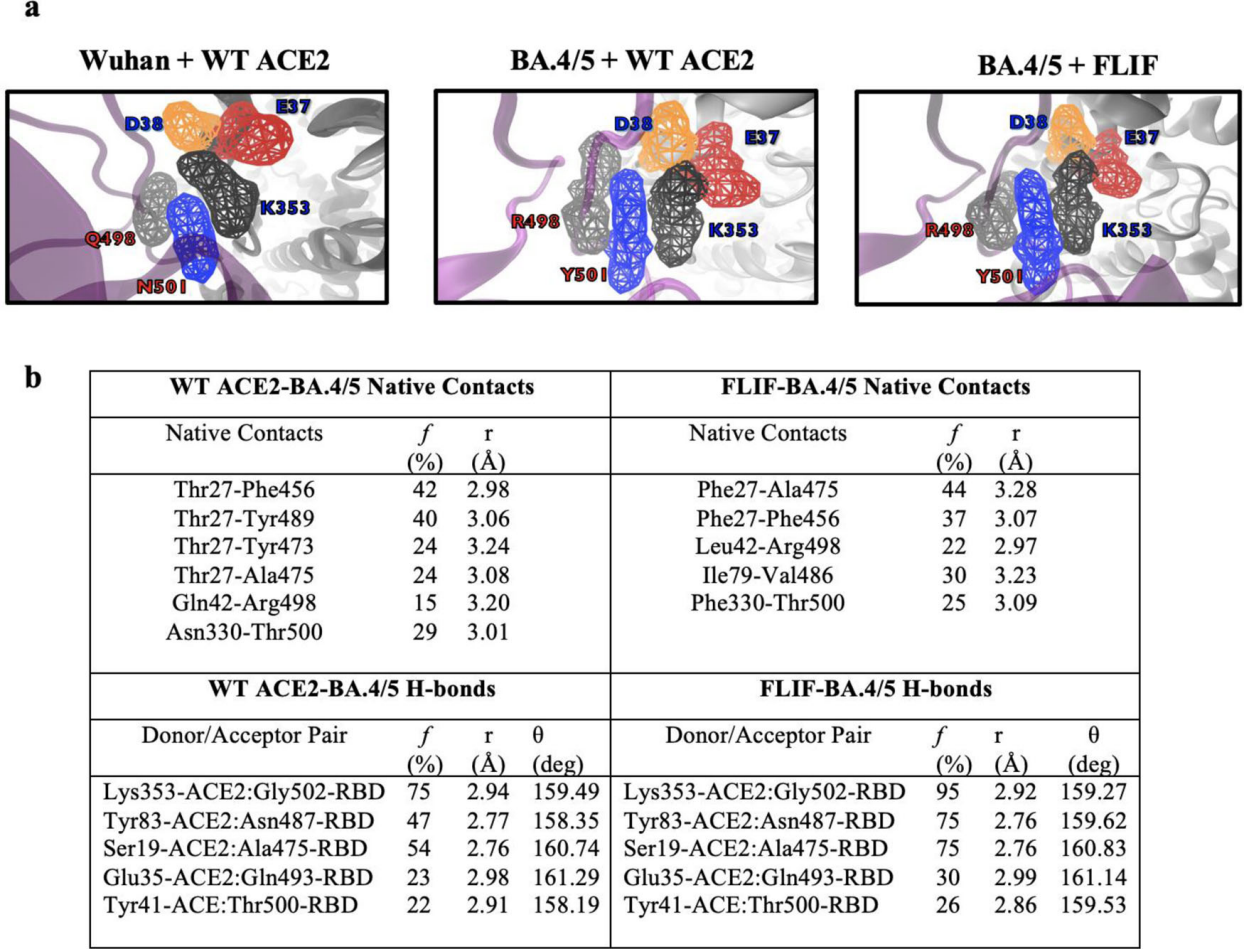
Rationale for affinity enhancement of FLIF to BA.4/5 using MD simulations. **a** Volumetric maps showing the 3D space occupied by key residues in the RBD: 501 (blue, Asn in Wuhan and Tyr in BA.4/5) and 498 (pale gray, Gln in Wuhan and Arg in BA.4/5). Interacting ACE2 residues are E37 (red), D38 (orange) and K353 (dark gray). Volumetric maps were created using VolMap Plugin in VMD^100^ with default parameters. **b** Native contacts of residues 27, 42, 79, and 330 in ACE2 for both wild type ACE2 and **FLIF** mutant with BA.4/5 RBD from MD simulations, along with H-bonds from wild type ACE2 and **FLIF** mutant with BA.4/5 RBD from MD simulations.

First, we analyzed the volumetric maps to show the three-dimensional space occupied by key residues Q498 and N501 in Wuhan-RBD, and R498 and Y501 in BA.4/5-RBD with both wild type and **FLIF** ACE2 during MD simulations (Fig. 7a).

In the simulation of Wuhan RBD, Q498-RBD orients itself towards N501-RBD while K353-ACE2 and Q498-RBD residues orient towards each other to make a “triangular” triad along with N501-RBD. Residues D38 and E37 in ACE2 face towards opposite directions. In comparison, in the BA.4/5 with WT ACE2 simulation, R498 and Y501 in RBD start to orient “vertically” while K353-ACE2 now moves away from R498 and Y501. In this simulation, D38 moves closer towards E37 in ACE2, and together these residues move towards K353-ACE2, orienting themselves away from R498-RBD and Y501-RBD (Fig. 7a). These MD simulations are in agreement with those conducted by Starr et al.^69^. Similarly, the BA.4/5 with **FLIF** mutant simulation show similar results; however, R498-RBD is now pointed more directly towards the **FLIF** mutant.

Next, we analyzed the hydrogen bond network and native contacts for the four mutations (T27F, Q42L, L79I, N330F) important in our **FLIF** decoy within 3.5 Å of BA.4/5 RBD (Fig. 7b). In the wild type ACE2 with BA.4/5 RBD simulation, we notice no hydrogen bonds were formed for ACE2 residues T27, Q42, L79, and N330 with BA.4.5 RBD. T27 is engaged in several hydrophobic contacts with RBD-F456, Y489, Y473, and A475, while Q42-ACE2 makes a contact with R498-RBD. There are no contacts for L79 within 3.5 Å of the BA.4/5 RBD and N330 makes a singular contact with RBD-T500. In comparison in our **FLIF**-BA.4/5 RBD simulation, the larger T27F mutation creates stronger hydrophobic interactions with RBD-A475 and pi-stacking interaction with RBD-F456, while the L42-ACE2 and R498-RBD interaction is extended with a shorter distance. In this simulation, the I79-ACE2 residue makes a hydrophobic contact with V486-RBD which is not seen with wild type ACE2 and suggests the L79I substitution with its isoleucine sec-butyl side chain orientation can improve hydrophobic packing with RBD-V486. In particular, the F486V mutation in the omicron subvariants severely reduces the activity of several antibodies, including AZD8895 (tixagevimab) in Evusheld, due to loss of the F486 aromatic interaction^67^. **FLIF** and ACE2 decoys more broadly are less susceptible to the impact of any singular mutation. Furthermore, in the **FLIF** mutant, the N330F mutation improves hydrophobic packing against the aliphatic portion of RBD-T500. When comparing BA.4/5 RBD with wild type ACE2 to BA.4/5 RBD with the **FLIF** mutant, there is an overall strengthening of the existing hydrogen bond network, likely due to improvement in hydrophobic packing which improves the overall shape complementarity of the ACE2-RBD interface. For example, occupancies of hydrogen bonding over the course of the simulations are strengthened for Lys353-ACE2:Gly502-RBD, Tyr83-ACE2:Asn487-RBD, Ser19-ACE2:Ala475-RBD, Glu35-ACE2:Gln493-RBD, and Tyr41-ACE:Thr500-RBD. Our MD simulations suggest that improved hydrophobic packing along with overall strengthening of the hydrogen bond network and the epistatic effect of the N501Y and Q498R mutations in the BA.4/5 RBD allows our **FLIF** decoy to bind more strongly to BA.4/5S RBD than wild type ACE2.

## Discussion

SARS-CoV-2 continues to mutate to evade monoclonal antibodies and antibodies elicited by vaccination. The most recent omicron subvariant, BA.5, is a perfect example showcasing substantial immune escape in comparison with earlier omicron variants^68,70,74^. Monoclonal antibodies and vaccination have been important in the fight against SARS-CoV-2 but omicron subvariants have rendered the majority of monoclonal antibodies in clinical use ineffective and vaccine immunity continues to wane, requiring updated boosters to remain effective against circulating variants^7,68^. As new variants continue to emerge with SARS-CoV-2 becoming endemic, there continues to be a great need for pan-coronavirus therapeutics that are resistant to mutational escape.

In contrast, guided by an orthogonal approach including computational protein design and free energy calculations, we designed a sACE2 decoy, **FLIF**, with picomolar affinity for delta of SARS-CoV-2 RBD and that remained effective at tightly binding omicron subvariants. Affinity engineered ACE2 decoys remain a promising strategy against SARS-CoV-2 due to their ability to outcompete native ACE2 receptors to neutralize the virus and their similarity to the native ACE2 receptor which make them effective against evolving variants of SARS-CoV-2. However, there is a balance that needs to be maintained between tight affinity and breadth. By engineering ACE2 decoys for tight affinity, breadth of neutralization may be compromised. We show that **FLIF** can potently neutralize previous SARS-CoV-2 variants as well as current circulating variants such as BA.5, providing evidence that **FLIF** is not over engineered at the expense of breadth. Furthermore, **FLIF** neutralized sarbecoviruses from both the SARS-CoV and SARS-CoV-2 clades showcasing its promise as a pan-coronavirus therapeutic and potential against developing SARS-related viruses that are risks for future zoonotic transmission.

Multiple groups have engineered ACE2 decoys with varying strategies. Glasgow et al.^33^ used an initial computational design approach and further refined their design using random mutagenesis and selection using yeast surface display, while others such as Chan et al.^31^, Higuchi et al.^32^, and Sims et al.^75^ used solely an experimental approach. Our group^29,30^ and others^34,39,76–79^ have employed a solely computational approach to designing ACE2 decoys. While a few of the groups have tested their computationally designed ACE2 decoys in vitro, only one group has tested a computationally designed ACE2 decoy in vivo against omicron BA.1^76^. Other computationally designed ACE2 decoys have also not yet been verified against newer circulating SARS-CoV-2 variants such as omicron BA.5. In fact, to the best of our knowledge, no ACE2 decoy (computationally or experimentally engineered) has been tested in vivo against newer omicron subvariants such as BA.5. Therefore, we verified the efficacy of our computationally designed ACE2 decoy, **FLIF**, against authentic omicron BA.5 in vitro and in vivo to answer the important question of whether engineered ACE2 decoys maintain efficacy, especially because BA.5 contains two unique mutations, F486V and L452R, not seen in previous omicron variants and that have caused extensive antibody escape^74^.

Moreover, other studies have investigated the in vitro and in vivo sensitivity of affinity matured ACE2 decoys against earlier SARS-CoV-2 variants such as BA.1^13,76,80^ but affinity matured ACE2 decoys have yet to be directly compared to soluble wild type ACE2, especially against the newer omicron variants. For the first time, we show that our computationally designed ACE2 decoy remains highly effective against the authentic SARS-CoV-2 omicron BA.5 strain in vitro and in vivo. In addition, when compared to the efficacy of wild type ACE2 (i.e., non-affinity enhanced sACE2) engineered ACE2 decoys such as **FLIF** provide a marked advantage at neutralizing omicron BA.5 in vitro and in vivo. A few wild type sACE2 decoys have shown to be effective against early circulating variants such as Wuhan in vivo^81,82^. However, our data suggests that moving forward affinity enhanced ACE2 decoys such as **FLIF** may be required to combat evolving SARS-CoV-2 variants. There are some limitations to our in vivo studies. Firstly, it is widely noted that omicron variants produce severely attenuated disease in mice and hamsters^83,84^. Thus, we were not able to investigate whether **FLIF** improves the pathogenicity of hamsters infected with more virulent variants of SARS-CoV-2 that cause rapid weight loss and severe lung pathology^37^. Second, **FLIF** was administered 2-h post-infection which may not mimic treatment of infection in humans. However, we have previously shown that ACE2 decoys 3N39v2 and sACE2_2_.v2.4 have therapeutic efficacy when administered 12-h, 24-h, and 2-days post-infection even against variants that produce severe lung pathology^32,37,80^.

In our ACE2 design, catalytic residues are left intact which likely means **FLIF** retains at least some of its catalytic activity. Many groups have opted to mutate the catalytic ACE2 residues to abolish peptidase activity arguing it might prevent unwanted off target effects^32–34,39,75,76,81,85^. However, recently Zhang et al.^80^ showed that the sACE2 catalytic activity improved the decoy’s therapeutic efficacy supporting a dual mechanism of action of competitive blocking of the SARS-CoV-2 S protein and turnover of ACE2 substrates associated with lung injury and inflammation. It is envisioned that **FLIF** will be more beneficial for treating lung injury from SARS-CoV-2 compared to catalytically attenuated ACE2 decoys. Furthermore, Yamaguchi et al.^86^ have shown that increasing ACE2-like enzymatic activity is a potential therapeutic strategy to alleviate COVID-19 related lung pathologies.

In conclusion, we used an orthogonal approach comprised of computational protein design, MD simulations, and free energy calculations to design an ACE2 mutant, **FLIF**, that exhibited tight binding to SARS-CoV-2 delta and omicron variants, displayed robust therapeutic utility against a broad range of SARS-CoV-2 variants and sarbecoviruses, and neutralized the dominant circulating variant worldwide, omicron BA.5, in vitro and in vivo. Orthogonal approaches combining computational and experimental methods remain promising for discovering small molecule and protein inhibitors of SARS-CoV-2^87,88^. Recently, Maschietto et al.^87^ used a computational approach to discover a valproate-coenzyme A conjugate that works allosterically to stabilize the RBDs in the trimeric “down” configuration to prevent binding to ACE2. The approach described herein emphasizes how computational methods have become sufficiently accurate for the design of therapeutics against viral protein targets and further shows the utility of engineered ACE2 decoys to remain effective against future SARS-CoV-2 variants.

## Methods

### Plasmids

Plasmid pcDNA3-sACE2-WT(732)-IgG1 (Addgene #154104; from N- to C-terminus, human ACE2 residues 1-732 fused to human IgG1 Fc) was used as a template for overlap extension PCR to introduce the **FLIF** mutations (T27F, Q42L, L79I, N330F). The pcDNA3.1(+) plasmids for mammalian cell expression of myc-tagged BA.2 omicron Spike and delta RBD-8h are previously described^37,38^. The pcDNA4TO plasmids for Spike with the ΔC19 (19 amino acids deleted from the C terminus) of SARS-CoV-2 variants (D614G, Delta, BA.1, BA.2, and BA4/5) and sarbecoviruses (SARS-CoV-1, PG-GD-1, PG-GX-P5L, RsSHO014, and WIV1) are previously described^13^.

### Flow cytometry

Expi293F cells (a suspension culture derivative of HEK293; Thermo Fisher Scientific) were cultured at 37 °C, 125 rpm, 8% CO_2_ in Expi293 Expression Medium (Thermo Fisher Scientific). Cells were transfected at 2 × 10^6^/ml using ExpiFectamine 293 (Thermo Fisher Scientific) with 500 ng per ml culture of pcDNA3-myc-S (BA.2 omicron). Cells were centrifuged (600 × *g*, 60 s) 24–28 h post-transfection and washed with Dulbecco’s phosphate buffered saline (PBS) containing 0.2% bovine serum albumin (BSA). Cells were incubated 30 min on ice with a serial dilution of sACE2-IgG1 in PBS-BSA. Cells were washed twice and resuspended for 30 min on ice in 1/250 anti-human IgG1-APC (clone M1310G05, BioLegend) and 1/100 anti-myc FITC (chicken polyclonal, Immunology Consultants Biology). Cells were washed twice, resuspended in PBS-BSA, and analyzed on a BD Accuri C6 using instrument software. The main cell population was gated by forward-side scattering (Supplementary Fig. S1). Binding of sACE2-IgG1 was measured based on the mean APC fluorescence intensity. Background fluorescence of cells incubated without sACE2-IgG1 was subtracted.

### Protein purification

The expression in Expi293F cells and purification of delta RBD-8h is previously described^37^. sACE2-IgG1 proteins were expressed in transiently transfected Expi293F cells. The ACE2 proteins are expected to be fully glycosylated, as described for ACE2 proteins produced in the very similar HEK293 cell line^89^. All the glycosylation sites were maintained with none of the FLIF mutations at *N*-glycosylation motifs. Expi293F cells were prepared at 2 × 10^6^/ml. Per ml of culture, 500 ng plasmid was mixed with 3 μg polyethylenimine (MW 25,000; Polysciences) in 100 μl OptiMEM (Gibco), incubated at room temperature for 20 min, and added to cells. Expifectamine Transfection Enhancers (Thermo Fisher Scientific) were added after 16–22 h. Culture was harvested after 6-7 days and clarified by centrifugation (600 × *g*, 20 min, 4 °C, followed by a high-speed spin at 18,000 × *g*, 25 min, 4 °C). Supernatant was incubated with KANEKA KanCapA 3G Affinity resin (AnaSpec) for 1-2 h at 4 °C. Resin was washed with PBS and proteins eluted with 60 mM sodium acetate pH 3.7. The eluate was neutralized by adding 1 M Tris base. The protein was separated on a Superdex 200 Increase 10/300 GL (Cytivia) size exclusion chromatography column equilibrated with PBS. Peak fractions were pooled, concentrated, and protein concentration determined by absorbance at 280 nm using calculated molar extinction coefficients for the monomeric mature polypeptides.

### BioLayer interferometry

sACE2_2_-IgG1 proteins were immobilized on anti-human IgG Fc biosensors (Sartorius) in assay buffer (10 mM HEPES pH 7.6, 150 mM NaCl, 3 mM EDTA, 0.05% polysorbate 20, 0.5% non-fat dry milk). Sensors were equilibrated in buffer for 30 s to establish baseline, then transferred to delta RBD-8h solution for 60 s and back to assay buffer for 300 s. Data were collected on an Octet RED96a and analyzed using instrument software (Sartorius) with a global fit 1:1 binding model.

### Pseudotyped virus neutralization assay

The neutralization assay using pseudoviruses is previously described^13^. Spike protein-expressing pseudoviruses with a luciferase reporter gene were prepared by transfecting plasmids (pcDNA4TO Spike-ΔC19, psPAX2 (Addgene #12260), and pLenti firefly) into LentiX-293T cells with Lipofectamine 3000 (Invitrogen). After 48 h, supernatants were harvested, filtered with a 0.45 μm low protein-binding filter (SFCA) and frozen at –80 °C. The 293T/ACE2 cells were seeded at 10,000 cells per well in 96-well plates. Pseudoviruses and 3-fold dilution series of therapeutic agents were incubated for 1 h, then these mixtures were added to 293T/ACE2 cells. After 1 h incubation, the medium was changed. At 48 h post infection, cellular expression of the luciferase reporter, indicating viral infection, was determined using ONE-Glo Luciferase Assay System (Promega). Luminescence was read on Infinite F200 pro system (Tecan). This assay was performed in 4 replicates and the non-linear regression curve was calculated using Prism version 9 (GraphPad Software).

### SARS-CoV-2 neutralization assay

Vero-TMPRSS2 were seeded at 80,000 cells in 24 well plates and incubated overnight. Cells were then infected with SARS-CoV-2 at MOI of 0.1 together with the protein. After 2 h, cells were washed with fresh medium and incubated with fresh medium for 22 h. Culture supernatants were collected for qRT-PCR.

### In vivo experiments

Four weeks-old male Syrian hamsters were purchased from SLC Japan. Syrian hamsters were anaesthetized by intraperitoneal administration of 0.75 mg kg^−1^ medetomidine (Meiji Seika), 2 mg kg^−1^ midazolam (Sandoz) and 2.5 mg kg^−1^ butorphanol tartrate (Meiji Seika) and challenged with 1.0 ×10^4^ PFU (in 60 μL) via intranasal routes. After 2 h post infection, recombinant proteins (20 mg kg^−1^) were dosed through intraperitoneal injection. On 5 days post infection, all animals were euthanized and lungs were collected for qRT-PCR. Animal experimentation protocols were approved by the Institutional Committee of Laboratory Animal Experimentation of the Research Institute for Microbial Diseases, Osaka University (approval number R02-08-0).

### Quantitative RT-PCR of in vivo samples

In the small animal experiments, total RNA of lung homogenates was isolated using ISOGENE II (NIPPON GENE). Real-time RT-PCR was performed with the Power SYBR Green RNA-to-CT 1-Step Kit (Applied Biosystems) using a AriaMx Real-Time PCR system (Agilent). The relative quantitation of target mRNA levels was performed by using the 2-ΔΔCT method. The values were normalized by those of the housekeeping gene, β-actin. The following primers were used: for β-actin; 5’-TTGCTGACAGGATGCAGAAG-3’ and 5’-GTACTTGCGCTCAGGAGGAG- 3’, 2019-nCoV_N2; 5’- AAATTTTGGGGACCAGGAAC -3’and 5’- TGGCAGCTGTGTAGGTCAAC -3’, IL-6; 5’- GGA CAATGACTATGTGTTGTTAGAA −3’and 5’- AGGCAAATTTCCCAATTGTATCCAG −3’, MIP1a; 5’- GGTCCAAGAGTACGTCGCTG −3’and 5’- GAGTTGTGGAGGTGGCAAGG −3’, CCL5; 5’- TCAGCTTGGTTTGGGAGCAA −3’and 5’- TGAAGTGCTGGTTTCTTGGGT −3’, CXCL10; 5’- TACGTCGGCCTATGGCTACT −3’and 5’- TTGGGGACTCTTGTCACTGG −3’.

### MD simulations

Conventional MD simulations were performed to calculate the binding enthalpy and to provide rationale for affinity enhancement of **FLIF** using the AMBER 20 package^90,91^. The MD simulations used to calculate binding enthalpy via MM/GBSA were performed using PDB: 6M0J^62^ and mutations for the **FFWF** and **FLIF** systems were introduced using the solution builder from CHARMM-gui^92^. BA.4/5 was modeled using PDB: 7ZF7^93^ which includes SARS-CoV-2 omicron BA.2 RBD in complex with ACE2. BA.4/5 specific mutations and ACE2 **FLIF** specific mutations were introduced using CHARMM-gui solution builder^92^. In both cases, systems were prepared using the CHARMM-gui solution builder^94^ with AMBER ff19SB force field for proteins^95^. The systems were fitted using a rectangular water box with a radius of 10 Å from the complex’s surface and solvated using a series of OPC water molecules^96^, which is the suggested water model to be used with ff19SB^95^. In order to mimic physiological conditions, 0.15 M NaCl ions were added using the Monte-Carlo ion placing method. In total, there were 319,512 total atoms, including ~75,000 OPC water molecules contained in a 137 Å × 137 Å × 137 Å simulation box. A steepest decent energy minimization was carried out using CPU for 5000 cycles and then the conjugate gradient algorithm was used for 5000 cycles. All systems were subjected to an equilibration period of 2 ns under (canonical ensemble) NVT conditions. To restrain each of the complexes during equilibration, a positional restraint of 1 kcal/mol was implemented. The temperature was set at 303.15 K and was maintained using Langevin dynamics^97^. In the production simulations used for binding enthalpy calculations, 100 ns simulations were conducted in replicates of four using an initial random velocity, while in the atomistic rationale for affinity enhancement one long 200 ns simulation was performed. Production MD simulations were performed under NPT conditions where the temperature was kept at 303.15 K and pressure at 1 atm to mimic experimental conditions. A friction coefficient, γ, of 1.0 ps^–1^ was used for the Langevin thermostat, and the pressure was held constant with the Monte Carlo barostat. Integration was performed using a leap-frog algorithm with a 2-fs time step. All bonds involving hydrogen atoms were constrained to their equilibrium values using SHAKE^98^. Periodic boundary conditions were applied to all simulations with a nonbonded cutoff of 10 Å and the particle-mesh-Ewald method^99^ was used to treat all long-range interactions. Figure 7a was created using the VolMap plugin in VMD v1.9.3^100^. Using the default parameters, the atomic densities observed over a grid, where the width of gaussian functions centered at each grid point bore widths equal to the atomic radii in each respective residue then weighted by atomic mass. Then, the double sum of these gaussian distributions over the grid points (and over the course of the MD simulation) are used to generate an isosurface map. The isosurface maps are rendered using an occupancy threshold of 0.5.

### MM/GBSA relative free-energy calculations

MM/GBSA free energy calculations were conducted as previously described^29^. Briefly, the MM/GBSA binding free energies were calculated from 125 independent frames using the last 50 ns from the 100 ns explicit-solvent MD simulations. The first 50 ns were discarded for equilibration. The Generalized born method, developed by Onufriev and company^101^, was set to igb = 5 to estimate the solvation energy. The radii were set to mbondi2 and the salt concentration was set to 0.15 M. Additionally, the dielectric constant of solvent and dielectric constant of solute were set to 78.5 and 1.0, respectively, which are Amber default and recommended values. The solvent-accessible surface area (SASA) was calculated using γ = 0.0072 kcal/mol/Å^2^ and β = 0.0 kcal/mol, respectively. The conformational entropy change is usually computed by normal-mode analysis on a set of conformational snapshots taken from MD simulations. In this case, contribution from entropy is neglected because of its large computational cost and low prediction accuracy. The binding enthalpy was calculated for all four 100 ns MD replicates and averaged.

### Absolute binding free-energy calculations

The Central Limit Free Energy Perturbation (CL-FEP) approach^45^ was used in all absolute binding free energy (ABFE) calculations. For calculations involving wild type RBD we used PDB: 6M0J^62^, while calculations involving omicron BA.1 and BA.2 used PDBs: 7WBP^51^ and 7ZF7^93^, respectively. For BA.4/5 RBD we used PDB: 7ZF7, which contains omicron BA.2 RBD in complex with ACE2, as a template and introduced the BA.4/5 specific RBD mutations using the CHARMM-gui web server^94^. **FLIF** specific ACE2 mutations were also introduced using the CHARMM-gui web server. The sampling was performed using MD simulations of the individual proteins (i.e., RBD only, ACE2 only, RBD-ACE2 complex, and bulk solvent only). Each of the individual proteins for each system was run for 300 ns (i.e., 3 replica simulations of 100 ns each) for a total of 900 ns each ABFE calculation. The simulation boxes and MD setup were obtained using the CL-FEP GUI web server (https://clfep.zmb.uni-due.de/). The proteins were sampled under harmonic wall restraints on their bound-state conformations which allows to focus the sampling on the most relevant states. A force constant value of 100 kcal/mol Å^2^ was used for the harmonic wall restraints on the RMSD for both the host (ACE2) and ligand (RBD). The maximum center of mass (COM) distance between the COMs of the ligand (RBD) and its binding site (ACE2) was set to 5 Å and a force constant for the COM was set to 50 kcal/mol Å^2^. Each system’s individual simulation boxes (i.e., RBD only, ACE2 only, RBD-ACE2 complex, and bulk solvent only) underwent 10,000 steps of minimization, 0.15 ns equilibration in NVT ensemble, and another 0.25 ns equilibration in NPT ensemble. The production simulations for each simulation box were conducted for three replicates of 100 ns each (total 300 ns of sampling time for each RBD only, ACE2 only, RBD-ACE2 complex, and bulk solvent only; 900 ns total each calculation). All simulations were performed using NAMD 2.14^102^ and the CHARMM36m^103^ force field using TIP3P water molecules^104^. All simulations were performed at 1 atm, 300 K, and 0.10 NaCl ionic concentration to mimic experimental binding assays. The pressure was controlled via Langevin dynamics^97^ and an electrostatic cut-off of 12 Å was used with the Particle Mesh Ewald method^99^ for the treatment of long-range interactions. CL-FEP analysis was also performed using the web server (https://clfep.zmb.uni-due.de/). The analysis was performed using ten checkpoints containing increasing fractions of the total energy samples. An oversampling ratio of osr = 3^45^ was used to bring the free energy variance to the level of (kT)^45^, and the second order cumulant estimator (C2)^45^ was used to evaluate the free energy change at each checkpoint. The final ABFE corresponds only to the average among the converged checkpoints. The error was obtained from running the CL-FEP analysis 10 runs (for WT ACE2) and 3 runs (for **FLIF**). All SD < 1 kcal/mol indicates the simulations are well converged^45^.

### Rosetta protein design

For all Rosetta simulations we used PDB: 6M0J which includes the X-ray crystal structure of SARS-CoV-2 RBD bound with ACE2 solved at 2.45 Å resolution^62^. Initially, the structure was relaxed with coordinate constraints on the backbone and side chain heavy atoms for 10 relaxation simulations. The lowest energy structure was subjected to Rosetta minimization without restraints utilizing the beta_nov16 energy function. To create a second-generation ACE2 decoy, we introduced the T27F and N330F mutations from **FFWF**^29^ in ACE2 using Rosetta. Using the Rosetta “Coupled Moves” flexible backbone design protocol^40^, we redesigned the local environment around the T27F and N330F mutations. ACE2 residues within 5 Å of heavy atoms on the RBD interface were allowed to be redesigned (except S19, K31, F27 and F330 in ACE2) to all amino acids besides cysteine, while RBD residues (plus S19, K31, F27 and F330 in ACE2) could change rotamer and/or backbone conformations (“repacking”) to accommodate the new mutation side chains. In addition, minimization was applied to the interface backbone and side chain torsion angles. The Rosetta “Coupled Moves” design protocol was repeated for 100 simulations and the top 10% of designs based on the lowest summed cross-interface pairwise interactions between RBD and ACE2 were selected for further evaluation and evaluated using WebLogo from UC Berkeley (https://weblogo.berkeley.edu/logo.cgi)^105^. Rosetta scripts are included in the supporting information.

### Statistics and reproducibility

Data analyses were performed using GraphPad Prism Version 9 software (GraphPad Software). Statistically significant differences between MM/GBSA calculations (Fig. 2a) were determined by ANOVA with Tukey’s post hoc test. Data are presented as the means $\pm \,$SD and $\pm$ SEM (see figure legends). The neutralization assay using pseudoviruses were conducted in 4 technical replicates. The neutralization assay using live SARS-CoV-2 omicron BA.5 virus were conducted in 3 technical replicates. Syrian hamster infection study using live SARS-CoV-2 omicron BA.5 virus were performed (*n* = 6 in control group and *n* = 3 in treatment group). The flow cytometry binding experiment (*n* = 3 biological replicates). MM/GBSA free energy values calculated using the average from 4 independent MD simulations. CL-FEP free energy values calculated using 3 replicate MD simulations for the individual proteins (RBD, ACE2, RBD-ACE2, and solvent). All experimental replication is described in the manuscript figure legends.

### Reporting summary

Further information on research design is available in the Nature Portfolio Reporting Summary linked to this article.

## Supplementary information

Supplementary Information

Reporting Summary

## Data Availability

All source data is available from the corresponding author upon reasonable request.
