# Supplementary material for: A computationally designed ACE2 decoy has broad efficacy against SARS-CoV-2 omicron variants and related viruses in vitro and in vivo
Source: Commun Biol. 2023 May 12;6:513. doi: 10.1038/s42003-023-04860-9 (PMC10177734; doi:10.1038/s42003-023-04860-9)
Supplement: Supplementary file 1 — Supplementary Information [file 42003_2023_4860_MOESM1_ESM.docx]

**Supporting information for:**

**A computationally designed ACE2 decoy has broad efficacy against SARS-CoV-2 omicron variants and related viruses in vitro and in vivo**

Brandon Havranek^1,2,3^, Graeme Walker Lindsey^4^, Yusuke Higuchi^5^, Yumi Itoh^6^, Tatsuya Suzuki^6^, Toru Okamoto^6^, Atsushi Hoshino^5^, Erik Procko^4,7^ and Shahidul M. Islam^3,8^*

^1^Department of Chemistry, University of Illinois at Chicago, Chicago, IL 60607, USA.

^2^Sidney Kimmel Medical College at Thomas Jefferson University, Philadelphia, PA 19107,

USA.

^3^ComputePharma, LLC., Chicago, IL, USA

^4^Department of Biochemistry, University of Illinois, Urbana, IL, 61801, USA.

^5^Department of Cardiovascular Medicine, Graduate School of Medical Science, Kyoto Prefectural University of Medicine, Kyoto 602-8566, Japan.

^6^Institute for Advanced Co-Creation Studies, Research Institute for Microbial Diseases, Osaka University, Osaka, Japan.

^7^Cyrus Biotechnology, Inc., Seattle, WA, USA

^8^Department of Chemistry, Delaware State University, Dover, DE, 19901, USA.

Correspondence*: email: sislam@desu.edu, Tel. 312-355-3767

**
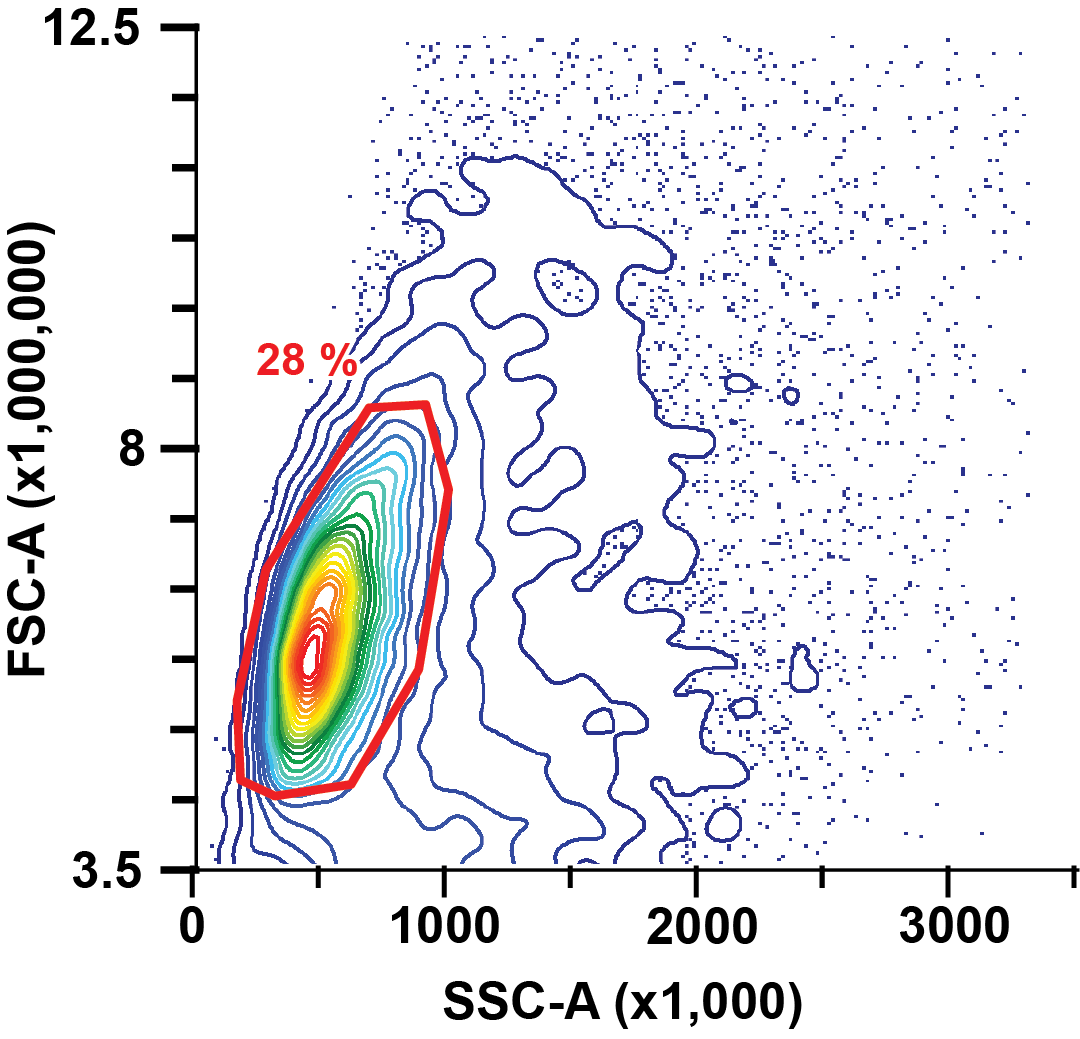
**

**Supplementary Figure S1. Gating strategy for flow cytometry analysis of sACE2-IgG1 bound to Spike-expressing cells.** Transiently transfected and sACE2-IgG1 / anti-human IgG APC stained Expi293F cells were tightly gated (red) by forward scatter (FSC) and side scatter (SSC) to select the main population of cells. A representative gated sample is shown. Mean APC fluorescence was then measured for the gated population.

**Rosetta command lines and bash scripts**

*Rosetta protein relaxation bash submission command:*

#!/bin/bash

#SBATCH -A che210078

#SBATCH -p standard

#SBATCH --nodes 1

#SBATCH --ntasks-per-node 1

#SBATCH --cpus-per-task 1

#SBATCH --time 24:00:00

#SBATCH -o myjob.relax_pdb.o

#SBATCH -e myjob.relax_pdb.e

#SBATCH -J ACE2_v2

srun /anvil/projects/x-che210078/rosetta.binary.linux.release 296/main/source/bin/relax.static.linuxgccrelease @relax_flags.txt -in:file:s 6m0j_final.pdb -database /anvil/projects/x-che210078/rosetta.binary.linux.release-296/main/database -relax:constrain_relax_to_start_coords -relax:coord_constrain_sidechains -out:suffix _relaxed -beta_nov16 -corrections::beta_nov16

@relax_flags.txt file:

-nstruct 10

*Rosetta protein minimization bash submission command:*

#!/bin/bash
#SBATCH -A che210078
#SBATCH -p standard
#SBATCH --nodes 1
#SBATCH --ntasks-per-node 1
#SBATCH --cpus-per-task 1
#SBATCH --time 1:00:00
#SBATCH -o myjob.minimize_pdb.o
#SBATCH -e myjob.minimize_pdb.e
#SBATCH -J ACE2_v2


srun /anvil/projects/x-che210078/rosetta.binary.linux.release-296/main/source/bin/minimize.static.linuxgccrelease -in:file:s 6m0j_final_relaxed_0003.pdb -database /anvil/projects/x-che210078/rosetta.binary.linux.release-296/main/database -out:suffix _min -beta_nov16 -corrections::beta_nov16

Redesign of ACE2 interface using T27F and N330F mutations from original **FFWF** mutant via Rosetta Coupled Moves protocol

*Redesign Rosetta Coupled Moves bash submission script:*

#!/bin/bash

#SBATCH -A che210078

#SBATCH -p standard

#SBATCH --nodes 1

#SBATCH --ntasks-per-node 1

#SBATCH --cpus-per-task 1

#SBATCH --time 24:00:00

#SBATCH -o myjob.red.o

#SBATCH -e myjob.red.e

#SBATCH -J ACE2v2

srun /anvil/projects/x-che210078/rosetta.binary.linux.release-296/main/source/bin/rosetta_scripts.static.linuxgccrelease -parser:protocol redesign.xml @redesign_flags.txt -database /anvil/projects/x-che210078/rosetta.binary.linux.release-296/main/database -out:suffix _FFWFv2

@redesign_flags.txt file:

-in
   -file
     -s 6m0j_final_relaxed_0003_min_0001.pdb
-packing
 -ex1
 -ex1aro
 -extrachi_cutoff 0
 -ex2
-number_ligands 0
-coupled_moves
 -initial_repack false
 -ligand_mode false
 -ligand_weight 0.0
-resfile 6m0j.resfile
-nstruct 100
-min_pack true
-beta_nov16
-overwrite
-mute core.util.prof
-mute core.io.database

Resfile “6m0j.resfile” used for redesign:

NATRO

start
19 A NATAA
24 A ALLAAxc
27 A NATAA
28 A ALLAAxc
30 A ALLAAxc
31 A NATAA
34 A ALLAAxc
35 A ALLAAxc
37 A ALLAAxc
38 A ALLAAxc
41 A ALLAAxc
42 A ALLAAxc
45 A ALLAAxc
79 A ALLAAxc
82 A ALLAAxc
83 A ALLAAxc
330 A NATAA
353 A ALLAAxc
354 A ALLAAxc
355 A ALLAAxc
357 A ALLAAxc
416 E NATAA
417 E NATAA
446 E NATAA
447 E NATAA
449 E NATAA
453 E NATAA
455 E NATAA
456 E NATAA
473 E NATAA
475 E NATAA
476 E NATAA
484 E NATAA
486 E NATAA
487 E NATAA
489 E NATAA
493 E NATAA
496 E NATAA
498 E NATAA
500 E NATAA
501 E NATAA
502 E NATAA
505 E NATAA

Rosetta XML Coupled Moves:

<ROSETTASCRIPTS>

<SCOREFXNS>
<ScoreFunction
name="beta"
weights="beta_nov16"/>
</SCOREFXNS>

<TASKOPERATIONS>
<ReadResfile
name="resfile"
filename="6m0j.resfile"/>
</TASKOPERATIONS>

<MOVERS>
<InterfaceAnalyzerMover
name="int_ddG"
scorefxn="beta"
fixedchains="A_E"/>

<MutateResidue
name="mutate_residue_1"
target="27A"
new_res="PHE"/>

<MutateResidue
name="mutate_residue_2"
target="330A"
new_res="PHE"/>

<MinMover
name="minimize"
scorefxn="beta"
chi="1"
bb="1"
tolerance="0.005"/>
 <RepackMinimize
name="repack_interface"
scorefxn_repack="beta"
scorefxn_minimize="beta"
repack_partner1="1"
repack_partner2="1"
design_partner1="0"
design_partner2="0"
interface_cutoff_distance="6.0"
repack_non_ala="1"
minimize_bb="1"
minimize_rb="1"
minimize_sc="1"
optimize_fold_tree="1"/>
 <CoupledMovesProtocol
name="coupled_moves"
task_operations="resfile"/>
</MOVERS>

<PROTOCOLS>

    <Add mover_name="mutate_residue_1"/>
    <Add mover_name="mutate_residue_2"/>


 <Add mover_name="repack_interface"/>
 <Add mover_name="coupled_moves" />
 <Add mover_name="repack_interface" />
 <Add mover_name="minimize"/>
<Add mover_name="int_ddG" />
</PROTOCOLS>

</ROSETTASCRIPTS>
