# Supplementary material for: A computationally designed ACE2 decoy has broad efficacy against SARS-CoV-2 omicron variants and related viruses in vitro and in vivo
Source: Commun Biol. 2023 May 12;6:513. doi: 10.1038/s42003-023-04860-9 (PMC10177734; doi:10.1038/s42003-023-04860-9)
Supplement: Supplementary file 2 — Reporting Summary [file 42003_2023_4860_MOESM2_ESM.pdf]

nature portfolio

Corresponding author(s): Shahidul M Islam

Last updated by author(s): Mar 8, 2023

Reporting Summary

Nature Portfolio wishes to improve the reproducibility of the work that we publish. This form provides structure for consistency and transparency in reporting. For further information on Nature Portfolio policies, see our Editorial Policies and the Editorial Policy Checklist.

Statistics

For all statistical analyses, confirm that the following items are present in the figure legend, table legend, main text, or Methods section.

n/a	Confirmed	
☐	☒	The exact sample size (n) for each experimental group/condition, given as a discrete number and unit of measurement
☐	☒	A statement on whether measurements were taken from distinct samples or whether the same sample was measured repeatedly
☐	☒	The statistical test(s) used AND whether they are one- or two-sided<br>*Only common tests should be described solely by name; describe more complex techniques in the Methods section.*
☒	☐	A description of all covariates tested
☐	☒	A description of any assumptions or corrections, such as tests of normality and adjustment for multiple comparisons
☐	☒	A full description of the statistical parameters including central tendency (e.g. means) or other basic estimates (e.g. regression coefficient) AND variation (e.g. standard deviation) or associated estimates of uncertainty (e.g. confidence intervals)
☐	☒	For null hypothesis testing, the test statistic (e.g. F, t, r) with confidence intervals, effect sizes, degrees of freedom and P value noted<br>*Give P values as exact values whenever suitable.*
☒	☐	For Bayesian analysis, information on the choice of priors and Markov chain Monte Carlo settings
☒	☐	For hierarchical and complex designs, identification of the appropriate level for tests and full reporting of outcomes
☒	☐	Estimates of effect sizes (e.g. Cohen's d, Pearson's r), indicating how they were calculated

Our web collection on statistics for biologists contains articles on many of the points above.

Software and code

Policy information about availability of computer code

Data collection	Binding affinity MD setup: CL-FEP web-server accessed at: https://clfep.zmb.uni-due.de/ and CL-FEP analysis performed at: https://clfep.zmb.uni-due.de/<br>MD simulations performed with: NAMD 2.14 (https://www.ks.uiuc.edu/Development/Download/download.cgi?PackageName=NAMD) and AMBER20 (https://ambermd.org/).<br>Rosetta Software suite for protein design (free for academic use): https://www.rosettacommons.org/software<br>CHARMM-GUI for MD setup: https://www.charmm-gui.org/<br>For the flow cytometry assay to determine binding between ACE2 and S BA.2, data were collected on a BD Accuri using the instrument software (CFlow version 1.0.264.15).<br>BLI kinetic binding data were collected on an Octet RED96a instrument (Sartorius).<br>For neutralization assay, luminescence was read on Infinite F200 pro system (Tecan).<br>For detection of viral RNA, real-time PCR was performed by a AriaMx Real-Time PCR system (Agilent).
Data analysis	Protein visualization: Pymol (open-source) (https://github.com/schrodinger/pymol-open-source), ChimeraX (https://www.rbvi.ucsf.edu/chimerax/) and VMD 1.9.3 (https://www.ks.uiuc.edu/Research/vmd/).<br>Sequence visualization from Rosetta simulations: WebLogo (https://weblogo.berkeley.edu/)<br>Prism version 9 (GraphPad Software) for figures with data and statistical analysis: (https://www.graphpad.com/)<br>Flow cytometry data were analyzed using CFlow version 1.0.264.15.<br>BLI kinetic binding data were analyzed using Octet Analysis Studio (Sartorius) using a global fit 1:1 binding model.

For manuscripts utilizing custom algorithms or software that are central to the research but not yet described in published literature, software must be made available to editors and reviewers. We strongly encourage code deposition in a community repository (e.g. GitHub). See the Nature Portfolio guidelines for submitting code & software for further information.

Data

Policy information about availability of data

All manuscripts must include a data availability statement. This statement should provide the following information, where applicable:

- Accession codes, unique identifiers, or web links for publicly available datasets
- A description of any restrictions on data availability
- For clinical datasets or third party data, please ensure that the statement adheres to our policy

All raw data sets will be provided by the corresponding author upon reasonable request. All other data generated or analyzed during this study is included in the published article or supporting information files. PDB accession codes for SARS-CoV-2 wild-type ACE2-RBD complex, SARS-CoV-2 Omicron BA.1 ACE2-RBD complex, and SARS-CoV-2 Omicron BA.2 ACE2-RBD complex are 6m0j, 7wbp, and 7zf7, respectively.

Human research participants

Policy information about studies involving human research participants and Sex and Gender in Research.

Reporting on sex and gender	n/a
Population characteristics	n/a
Recruitment	n/a
Ethics oversight	n/a

Note that full information on the approval of the study protocol must also be provided in the manuscript.

Field-specific reporting

Please select the one below that is the best fit for your research. If you are not sure, read the appropriate sections before making your selection.

☒ Life sciences ☐ Behavioural & social sciences ☐ Ecological, evolutionary & environmental sciences

For a reference copy of the document with all sections, see nature.com/documents/nr-reporting-summary-flat.pdf

Life sciences study design

All studies must disclose on these points even when the disclosure is negative.

Sample size	No statistical methods were used to predetermine the sample size. The sample sizes were based upon experimental feasibility and sample availability.
Data exclusions	No data were excluded.
Replication	Neutralization assay using pseudoviruses were conducted in 4 technical replicates. Neutralization assay using live SARS-CoV-2 omicron BA.5 virus were conducted in 3 technical replicates. Syrian hamster infection study using live SARS-CoV-2 omicron BA.5 virus were performed (n = 6 in control group and n = 3 in treatment group). Flow cytometry experiment (n = 3 biological replicates). MM/GBSA free energy values calculated using the average from 4 independent MD simulations. CL-FEP free energy values calculated using 3 replicate MD simulations for the individual proteins (RBD, ACE2, RBD-ACE2, and solvent). All experimental replication is described in the manuscript legends.
Randomization	Animals and samples were randomly sorted into experimental groups.
Blinding	The investigators were not blinded to group allocation for data collection and analysis for any experiment or computational work conducted in this study. The hamster experiments were age and sex-controlled.

Reporting for specific materials, systems and methods

We require information from authors about some types of materials, experimental systems and methods used in many studies. Here, indicate whether each material, system or method listed is relevant to your study. If you are not sure if a list item applies to your research, read the appropriate section before selecting a response.

Materials & experimental systems

n/a	Involved in the study	
☐	☒	Antibodies
☐	☒	Eukaryotic cell lines
☒	☐	Palaeontology and archaeology
☐	☒	Animals and other organisms
☒	☐	Clinical data
☒	☐	Dual use research of concern

Methods

n/a	Involved in the study	
☒	☐	ChIP-seq
☐	☒	Flow cytometry
☒	☐	MRI-based neuroimaging

Antibodies

Antibodies used	anti-human IgG-APC (clone M1310G05, BioLegend, Cat. 410712)<br>anti-MYC-FITC (chicken polyclonal, Immunology Consultants Laboratory, Cat. CMYC-45F)
Validation	Antibodies are commercially supplied and validated by the vendors.

Eukaryotic cell lines

Policy information about cell lines and Sex and Gender in Research

Cell line source(s)	Expi293F (ThermoFisher)<br>Lenti-X 293T (Clontech, 632180)<br>Vero-TMPRSS2 was obtained from the JCRB cell bank in JAPAN.
Authentication	Cell lines were authenticated by commercial vendors and by morphological appearance.
Mycoplasma contamination	The cell lines were tested for potential mycoplasma contamination and confirmed that they are mycoplasma negative.
Commonly misidentified lines<br>(See ICLAC register)	No commonly misidentified cell lines were used in this study.

Animals and other research organisms

Policy information about studies involving animals; ARRIVE guidelines recommended for reporting animal research, and Sex and Gender in Research

Laboratory animals	This study used 4-week-old male Syrian hamsters.
Wild animals	This study did not involve wild animals.
Reporting on sex	Only male Syrian hamsters were utilized in this study. Male hamsters present with a greater susceptibility to SARS-CoV-2 infection and experience more severe symptoms of COVID-19 (https://doi.org/10.1038/s41392-021-00552-0).
Field-collected samples	This study did not involve field-collected samples.
Ethics oversight	Animal experimentation protocols were approved by the Institutional Committee of Laboratory Animal Experimentation of the Research Institute for Microbial Diseases, Osaka University (approval number R02-08-0).

Note that full information on the approval of the study protocol must also be provided in the manuscript.

Flow Cytometry

Plots

Confirm that:

☒ The axis labels state the marker and fluorochrome used (e.g. CD4-FITC).

☒ The axis scales are clearly visible. Include numbers along axes only for bottom left plot of group (a 'group' is an analysis of identical markers).

☒ All plots are contour plots with outliers or pseudocolor plots.

☒ A numerical value for number of cells or percentage (with statistics) is provided.

Methodology

Sample preparation	To analyze Spike/ACE2 interactions, Expi293F cells were transfected with full-length, myc-tagged Spike, washed post-

Sample preparation	transfection with PBS containing 0.2% BSA, incubated with soluble ACE2-IgG1 proteins, and then stained with anti-myc-FITC and anti-human IgG1-APC as described in Methods.
Instrument	BD Accuri
Software	CFlow version 1.0.264.15
Cell population abundance	Transfected Expi293F cells positive for the myc tag were analyzed. No cells were sorted.
Gating strategy	Cells were gated by FSC/SSC for the main cell population.

☒ Tick this box to confirm that a figure exemplifying the gating strategy is provided in the Supplementary Information.
